# Manganese powder promoted highly efficient and selective synthesis of fullerene mono- and biscycloadducts at room temperature

**DOI:** 10.1038/srep13920

**Published:** 2015-09-09

**Authors:** Weili Si, Xuan Zhang, Shirong Lu, Takeshi Yasuda, Naoki Asao, Liyuan Han, Yoshinori Yamamoto, Tienan Jin

**Affiliations:** 1 WPI-Advanced Institute for Materials Research (WPI-AIMR), Tohoku University, Sendai 980-8577, Japan; 2 Photovoltaic Materials Unit, National Institute for Materials Science, Tsukuba 305-0047, Japan; 3 State Key Laboratory of Fine Chemicals, Dalian University of Technology, Dalian 116012, China

## Abstract

Discovery of an efficient, practical, and flexible synthetic method to produce various important electron acceptors for low-cost organic photovoltaics (OPVs) is highly desirable. Although the most commonly used acceptor materials, such as PC_61_BM, PC_71_BM, IC_60_BA, bisPC_61_BM have been proved to be promising for the OPVs, they are still very expensive mainly due to their low production yields and limited synthetic methods. Herein, we report an unprecedented and innovative synthetic method of a variety of fullerene mono- and biscycloadducts by using manganese powder as a promotor. The reaction of fullerenes with various dibromides proceeds efficiently and selectively under very mild conditions to give the corresponding cycloadducts in good to excellent yields. The combination of manganese power with DMSO additive is crucial for the successful implementation of the present cycloaddition. Notably, the standard OPV acceptors, such as PCBMs, have been obtained in extraordinarily high yields, which cannot be achieved under the previously reported methods.

Functional fullerenes have been used broadly as excellent n-type semiconductors in solution processable organic electronics^1^,^2^,^3^,^4^,^5^, especially as the unique electron acceptors for organic photovoltaics (OPVs) due to their significant increase in solubility while preserving certain electronic and optical properties of pristine fullerenes^6^,^7^,^8^. At present, the state-of-the-art of OPVs have an overall power conversion efficiency approaching 10%^9^,^10^,^11^ based on newly developed low bandgap electron donors and [6,6]-phenyl-C_61_-butyric acid methyl ester (PC_61_BM) or its C_70_ analogue PC_71_BM as an electron acceptor^12^,^13^,^14^ which are the most well-demonstrated benchmark acceptors for testing new donor materials in terms of their miscibility, solubility, and high electron mobility^15^,^16^. Recently, many endeavors on development of new functional fullerenes have been made to improve the efficiencies of OPVs^17^,^18^,^19^,^20^,^21^,^22^,^23^,^24^,^25^,^26^,^27^ and it was found that the bisfunctional fullerenes with up-shifted LUMOs, such as indene-C_60_-bisadduct (IC_60_BA)^19^,^20^, bisPC_61_BM^21^ and bis-*o*-quinodimethane C_60_ (*o*-QDMC_60_)^22^,^23^,^24^ showed higher open circuit voltages and hence improved OPV efficiencies. In this context, it is expected that the efficiency could be further improved that may expedite the practical application of the OPVs in the next few years. To achieve low-cost OPVs, synthesis of OPV materials in a simple, practical process with a high production yield is one of the important strategies^28^. However, the standard acceptors PCBM, ICBA, and their analogues are still very expensive due to low yields, low selectivities, and harsh synthetic conditions. For example, PC_61_BM was prepared by a one-pot reaction over two-steps in 58% yield through the reaction of C_60_ with methyl 4-benzoylbutyrate *p*-tosylhydrazone at 70 °C followed by isomerization of the resulting [5,6]PC_61_BM to PC_61_BM at 180 °C (Fig. 1a)^13^. IC_60_BA was synthesized in 34% yield along with the formation of the monoadduct IC_60_MA in 25% yield in the reaction of C_60_ with indene at 180 °C (Fig. 1a)^19^. Therefore, development of an innovative, practical synthetic method for those important fullerene acceptors under mild conditions with high production yields is highly desirable.

Recently, we have been interested in development of new and efficient fullerene functionalizations under mild conditions toward application in OPVs^29^,^30^,^31^,^32^,^33^,^34^,^35^,^36^. Various fullerene functionalizations for synthesis of monosubstituted hydrofullerenes, monocycloadducts, single-bonded fullerene dimers, 1,4-disubstituted fullerenes have been developed in the presence of transition metal catalysts or oxidant via the formation of the fullerene monoradical as an active species^37^. These results led us to challenge the innovative, flexible synthetic method for those most common fullerene acceptors in very high yields. Herein, we report an unprecedented, highly efficient, and selective manganese powder-promoted bis- and monocycloaddition of C_60_ (or C_70_) with various alkyldibromides at room temperature for constructing carbocycle-fused fullerenes with various ring sizes (Fig. 1b). Notably, the present cycloaddition afforded PC_61_BM and PC_71_BM in excellent yields of >90%, as well as IC_60_BA and bisPC_61_BM in 75% and 92% yields, respectively, which cannot be achievable under the previously reported methods.

## Results

### Optimization of the reaction conditions

As aforementioned, we have reported that the CoCl_2_dppf catalyst combined with a Mn reductant in *o*-dichlorobenzene (ODCB) promoted the monocycloaddition of C_60_ with active dibromides efficiently at room temperature to form the fullerene monocycloadducts in a high selectivity^30^. It was noted that the efforts to synthesize the biscycloadducts under the Co-catalyzed standard conditions even using an excess amount of dibromides were failed. Further investigations of the reaction conditions for the selective biscycloaddition of C_60_ with 1,2-bis(bromomethyl)benzene (**1a**, 4 equiv) are summarized in Table 1.

The reaction with Mn powder (9 equiv) in ODCB did not produce any desired cycloadducts (entry 1). We have previously demonstrated that the use of polar cosolvents with ODCB remarkably enhanced the fullerene monoradical generation and stability^33^,^34^,^35^,^36^, which led us to examine various polar cosolvents. To our delight, the reaction proceeded smoothly in a mixture of DMSO/ODCB with Mn powder at room temperature, affording the corresponding biscycloadduct **2a** (*o*-QDMC_60_) in 60% yield together with a 20% yield of the multicycloadduct **3a** (entry 2). The reaction did not take place without using Mn powder in this solvent system (entry 3). Other cosolvents were also tested in the presence of Mn powder. It was found that the use of DMF as a cosolvent exhibited a much higher activity, yielding the multiadducts predominantly without formation of both bis- and monocycloadducts (entry 4), while other polar solvents such as CH_3_CN, THF, and EtOH were totally inactive (entries 5–7). Notably, the decrease in amounts of Mn powder (3 equiv) and dibromide **1a** (2.2 equiv) in ODCB/DMSO solvent systems hampered the formation of multiadducts under a prolonged reaction time (47 h), resulting in **2a** in an 85% isolated yield (entry 8). Other metals were also tested instead of Mn powder. In contrast to the high activity of Mn(0), other Mn salts such as MnO_2_, Mn(OAc)_3_•2H_2_O^38^, and MnCl_2_•4H_2_O having a higher oxidation state were totally ineffective (entries 9–11). Zinc and iron powders which have been employed as reductants successfully in our previous cobalt catalysis^29^ showed a moderate activity, in which Zn powder could promote the present biscycloaddition to give **2a** in 55% yield, while Fe powder showed a relatively lower reactivity to produce the monocycloadduct **3a** in 54% yield without formation **2a** (entries 12 and 13). The reactions using other zero-valent metals, such as Mg, Cu, Pd/C, and Raney Ni did not show any activity for promoting the present cycloaddition (entries 14–17). Overall, it is concluded that the use of Mn(0) powder in DMSO/ODCB solvent systems is crucial for the selective formation of the fullerene biscycloadduct **2a** in a high chemical yield.

### Synthesis of biscycloadducts

Under the optimized conditions, various alkyl dibromides have been examined to study the substrate scope and selectivity of the present biscycloaddition (Fig. 2). All the reactions were monitored by HPLC and the corresponding products were isolated by silica gel chromatography. It is noted that very small amounts of the monoadducts and the recovered C_60_ were observed in every reaction, and the major by-products were multiadducts whose yields did not show due to some overlapping peaks with bisadducts in HPLC chromatogram. The new structures of biscycloadducts containing a mixture of isomers were determined by ^1^H and ^13^C NMR spectra as well as the high resolution mass.

**Figure 1:**
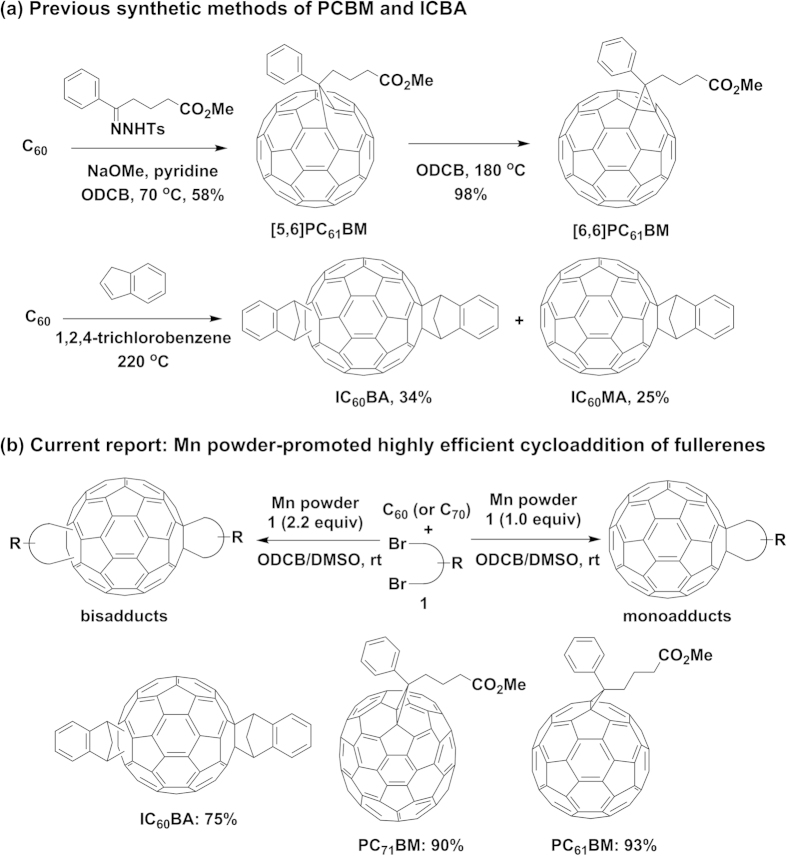
Synthesis of the most common OPV acceptors. (**a**) The previous synthetic methods for PCBM and ICBA under thermal conditions. (**b**) Our new method of Mn powder-promoted synthetic method for achieving high yields of mono- and biscycloadducts including PC_61_BM, PC_71_BM, and IC_60_BA.

**Figure 2:**
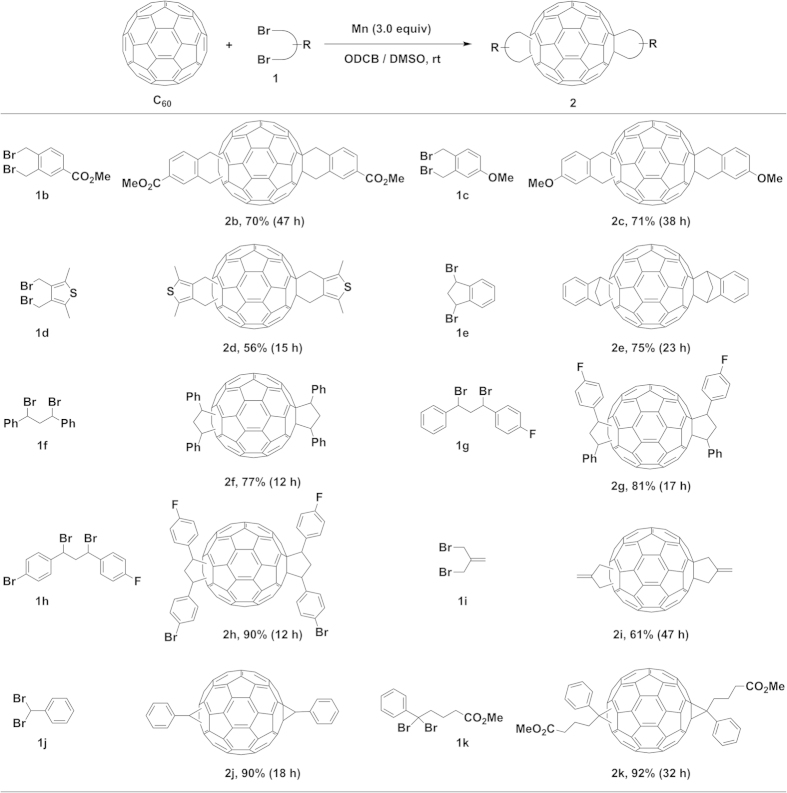
Mn-promoted biscycloaddition of C_60_ with various alkyl dibromides. Reaction conditions: C_60_ (0.03 mmol), Mn (0.09 mmol, 3 equiv), dibromides (**1**, 2.2 equiv), DMSO (0.3 ml), ODCB (4 ml), under an argon atmosphere at room temperature. The isolated yields are shown after silica gel chromatography.

Firstly, we tested various bis(bromomethyl)arenes to obtain the corresponding bisadducts fused with a 6-membered ring. 1,2-Bis(bromomethyl)benzenes **1b** and **1c** having a ester or a methoxy group on the phenyl ring were tolerated under the present conditions, furnishing the corresponding biscycloadducts **2b** and **2c** in 70% and 71% yields, respectively. The reaction is also compatible with the heteroaryl-incorporated dibromide, 3,4-bis(bromomethyl)-2,5-dimethylthiophene (**1d**), affording the corresponding bisadduct **2d** in 56% yield. The reaction of C_60_ with 1,3-dibromo-2,3-dihydro-1*H*-indene (**1e**) produced IC_60_BA (**2e**) in 75% yield which is much higher than that using the previously reported method (34%, Fig. 1a)^19^. It is noted that the scale-up reaction of C_60_ (108 mg) with **1e** did not show significant decrease in the efficiency, giving IC_60_BA (**2e**) in a 72% isolated yield after 26 h at room temperature (Figure S3). The reaction is also applicable to the construction of the bisadducts fused with a 5-membered ring, which could not be prepared by the previously reported methods^22^,^23^,^24^. The reactions of C_60_ with 1,3-dibromo-1,3-diphenylpropane (**1f**), and its derivatives **1g** and **1h** having Br and F substituents on the phenyl ring showed a high reactivity, producing the corresponding bisadducts **2f–h** in good to high yields in short reaction times. Again, it was confirmed that the scale-up reaction of C_60_ (108 mg) with **1f** produced a 75% yield of **2f** under a prolonged reaction time (45 h) (Figure S4). The allylic dibromide such as 3-bromo-2-(bromomethyl)prop-1-ene (**1i**) is also a suitable substrate for the present cycloaddition, giving the corresponding bisadduct **2i** in 61% yield. Interestingly, the reaction of C_60_ with (dibromomethyl)benzene (**1j**) gave the 3-membered ring fused bisadduct **2j** in 90% yield. This result encouraged us to synthesize bisPCBM which has also been applied as a promising acceptor in OPVs^21^. The corresponding dibromide reactant, methyl 5,5-dibromo-5-phenylpentanoate (**1k**) was prepared in 90% yield by the dibromination of methyl 5-phenylpentanoate with NBS in the presence of a catalytic amount of AIBN at reflux (Figure S1). Remarkably, the biscycloaddition of C_60_ with **1k** under the standard conditions produced the desired bisPC_61_BM (**2k**) in 92% yield. It is worthy to note that bisPC_61_BM is generally obtained as a by-product during the preparation of PC_61_BM^21^.

### LUMO energy levels of biscycloadducts

It was reported that 56π-biscycloadducts, such as IC_60_BA and bisPC_61_BM exhibited a significantly improved open circuit voltage (*V*_oc_) owing to its much higher LUMO energy than that of PCBM^19^,^20^,^21^. We have measured the LUMO energy levels of the selected new biscycloadducts by cyclic voltammetry (CV). Biscycloadducts **2b** and **2c** having a ester or a methoxy group on the phenyl ring showed high LUMOs of −3.48 and −3.41 eV, respectively, which are higher than that of PC_61_BM (−3.60 eV) and comparable with that of IC_60_BA (**2e**, −3.43 eV). The 5-membered ring-fused bisadduct **2i** shows a slightly higher LUMO energy level (−3.40 eV) compared with IC_60_BA (**2e**). Since the *V*_oc_ of bulk heterojunction solar cells has an association with the energy difference between the LUMO of acceptor and the HOMO of donor^39^, it is expected that the new biscycloadducts possessing higher LUMO energies should be potential acceptor candidates for OPVs.

### Synthesis of monocycloadducts

**Figure 3:**
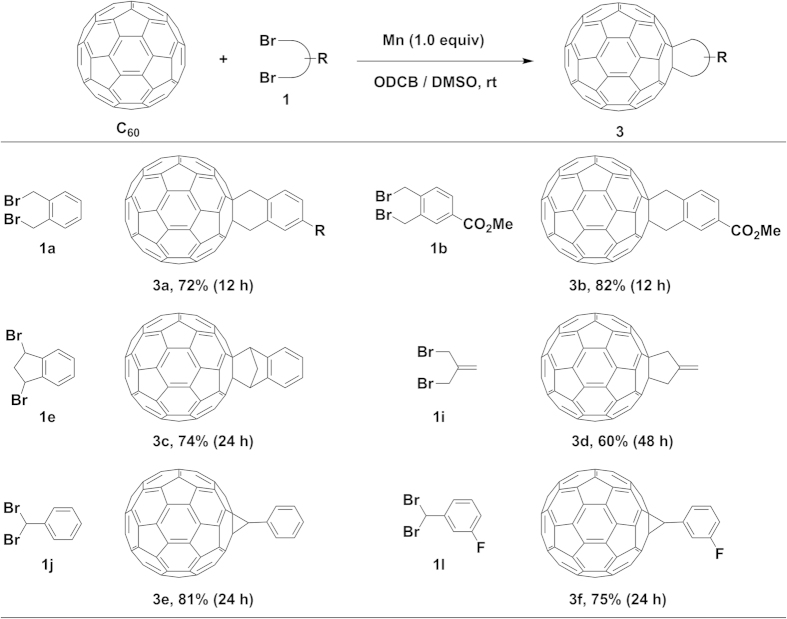
Mn powder-promoted selective monocycloaddition of C_60_ with alkyl dibromides. Reaction conditions: C_60_ (0.03 mmol), Mn (1.0 equiv), **1** (1.0 equiv), DMSO (0.4 mL), ODCB (4 mL), under an argon atmosphere at room temperature. The Isolated yields are shown after silica gel chromatography.

**Figure 4:**
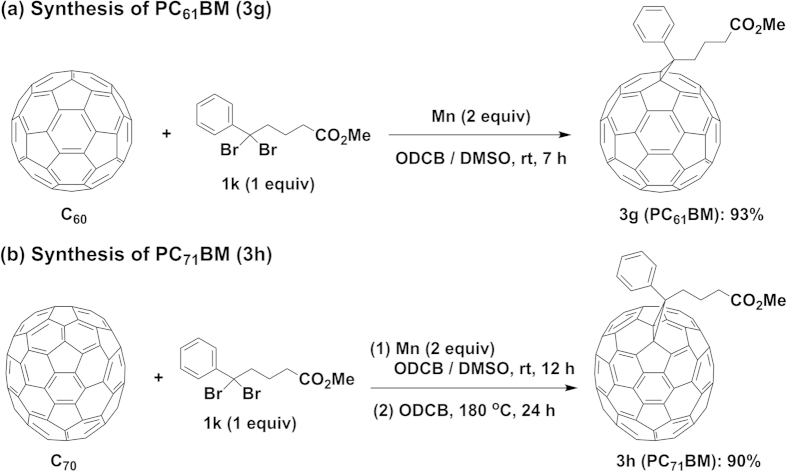
Synthesis of PC_61_BM and PC_71_BM. Reaction conditions: C_60_ or C_70_ (0.03 mmol), Mn (2.0 equiv), **1k** (1.0 equiv), DMSO (0.4 mL), ODCB (4 mL), under an argon atmosphere at room temperature. The Isolated yields are shown after silica gel chromatography. (**a**) Synthesis of PC_61_BM. (**b**) Synthesis of PC_71_BM. The products obtained from the reaction at rt for 12 h, were further heated at 180 °C for 24 h.

Inspired by the successful biscycloaddition of C_60_ with Mn powder, we further extended the present method to the selective monocycloaddition. To our delight, the monocycloadducts could be obtained at room temperature in good to excellent yields with a wide substrate scope by simply decreasing the amounts of Mn powder (1 equiv) and dibromides (1 equiv) as shown in Fig. 3. The reaction of C_60_ with bis(bromomethyl)benzenes **1a** and **1b** in the presence of Mn powder produced the 6-membered ring-fused monocycloadducts **3a** and **3b** in 72% and 82% yields, respectively, with a small amount of the recovered C_60_. The indene-monoadduct **3c** can be prepared in 74% yield under the standard conditions using 1,3-dibromo-2,3-dihydro-1*H*-indene (**1e**) as a dibromide source. Monocycloaddition of 3-bromo-2-(bromomethyl)prop-1-ene (**1i**) with C_60_ furnished the corresponding 5-membered ring fused monocycloadduct (**3d**) in 60% yield. (Dibromomethyl)benzenes **1j** and **1l** underwent the selective monocycloaddition with C_60_ smoothly to afford the corresponding cyclopropyl-fused monoadducts **3e** and **3f** in 81% and 75% yields, respectively. Surprisingly, when **1k** was used as a dibromide reactant, PC_61_BM (**3g**) was obtained in a very high yield of 93% using 2 equiv of Mn powder after 7 h at room temperature (Fig. 4a). The large-scale reaction of C_60_ (200 mg) with **1k** under the identical conditions produced PC_61_BM (**3g**) in a slightly lower yield of 88% (12 h, Figure S6), demonstrating that the efficiency of the present method stays high at large scale. It is noted that a 38% yield of PC_61_BM could be obtained under our previously reported Co-catalyzed reaction conditions^30^ after 24 h along with the recovered C_60_ in 54% yield (Figure S7). However, under the present reaction conditions, the cycloaddition of C_70_ with **1k** produced a mixture of [6,6]- and [5,6]-isomers after 12 h. Subsequently, the mixture purified by silica gel chromatography was further heated at 180 °C in ODCB for 24 h to give the corresponding [6,6]-isomers PC_71_BM (**3h**) in 90% yield over two steps (Fig. 4b). Noted that the signals for three methoxy groups in the ^1^H NMR spectrum indicated that the ratio of three isomers in PC_71_BM was 38:46:16, which is different with that observed from the reported method^14^.

## Discussion

In general, the biscycloadduct *o*-QDMC_60_ (**2a**) and the monocycloadduct **3a** can be prepared through the Diels-Alder reaction of C_60_ with *o*-quinodimethane generated *in situ* from 1,2-bis(bromomethyl)benzene (**1a**) at high temperatures^22^,^23^,^24^,^40^. The question should be whether the present cycloaddition proceeds through the formation of *o*-quinodimethane from **1a** by Mn powder, which was ruled out based on the good reactivity of other dibromides **1f–k** which could not form the *o*-quinodimethane-like intermediates. The other possible mechanism is the formation of a fullerene radical anion by metal reductants^41^ followed by the reaction with dibromides^42^. However, this pathway seems to be unlikely because we found that the reaction of C_60_ and H_2_O in the absence of dibromides under otherwise the standard conditions did not produce any hydrogenated C_60_-adducts including the expected dihydrofullerene (C_60_H_2_), which is different from the Zn-mediated monoalkylation of C_60_ with alkylbromides as reported by Meier *et al.*^43^ Moreover, we found that the reaction of the dibromide **1a** and benzaldehyde with Mn powder in the absence of C_60_ under otherwise the standard conditions did not afford any products and the starting substrates were recovered quantitatively, implying that the formation of the organomanganese reagents^44^ cannot be accounted for the present cycloaddition. It is also noted that the existence of a small amount of air slows the reaction rate and much amounts of Mn powder and dibromides are required with prolonged reaction times. At present, although the present cycloaddition mechanism is yet to be determined, we assume that the reaction should be initiated by the electron transfer among C_60_, dibromides, and Mn powder. The detailed mechanistic studies will be reported in due course.

In conclusion, we have described a novel and highly efficient manganese powder-promoted fullerene cycloaddition with various alkyl dibromides. The reaction procedure is flexible, practical, and mild, which produces a variety of new and known fullerene cycloadducts with various carbocycle sizes in high chemical yields and high mono- and biscycloaddition selectivities. Notably, we have succeeded for the first time in synthesis of the most common OPV acceptors, such as PC_61_BM, PC_71_BM, IC_60_BA, and bisPC_61_BM in good to excellent yields. The combination of manganese powder with the DMSO cosolvent is vital for the implementation of the present cycloaddition sufficiently. Our method not only provides an efficient, low-cost, and general approach for the formation of the important functional fullerenes, but also may boost the realization of the practical application of OPVs.

## Methods

### Scale-up procedure for synthesis of IC_60_BA (2e)

To a mixture of 1,2-dichlorobenzene (20 mL), DMSO (1.5 mL), C_60_ (108 mg, 0.15 mmol), and Mn (24.7 mg, 0.45 mmol, 3 equiv.) was added methyl 1,3-dibromo-2,3-dihydro-1H-indene (**1e**, 91 mg, 0.33 mmol, 2.2 equiv.) under an argon atmosphere. The reaction mixture was stirred at room temperature for 26 h to give a dark brown solution. After monitoring with HPLC, the mixture was purified directly by silica gel chromatography using hexane/CS_2_ (1/1) as eluents. The product was washed with methanol and dried to afford the corresponding IC_60_BA (**2e**) in 72% yield (103 mg).

### Scale-up procedure for synthesis of PC_61_BM (3g)

To a mixture of 1,2-dichlorobenzene (40 mL), DMSO (4 mL), C_60_ (200 mg, 0.277 mmol), and Mn (30.4 mg, 0.554 mmol, 2 equiv) was added methyl 5,5-dibromo-5-phenylpentanoate (**1k**, 97 mg, 0.277 mmol, 1 equiv) under an argon atmosphere in glove box. The reaction mixture was stirred at room temperature for 12 h to give a dark brown solution. After monitoring with HPLC, the mixture was purified directly by silica gel column chromatography using toluene as an eluent. The isolated product was washed with methanol and dried to afford PC_61_BM (**3g**) in 88% yield (222 mg).

## Additional Information

**How to cite this article**: Si, W. *et al.* Manganese powder promoted highly efficient and selective synthesis of fullerene mono- and biscycloadducts at room temperature. *Sci. Rep.* **5**, 13920; doi: 10.1038/srep13920 (2015).

## Supplementary Material

Supplementary Information

## Figures and Tables

**Table 1:** Investigation of the reaction conditions for synthesis of biscycloadduct 2a (*o*-QDMC60).

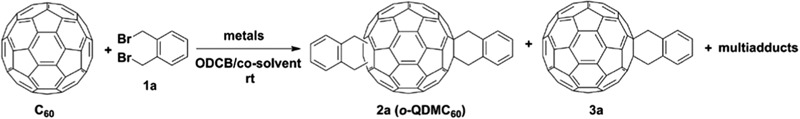						
**Entry**	**Metal (equiv)**	**Co-solvent**	**2a [%]**†	**3a [%]**†	**Multiadducts [%]**†	**Recovery of C**_**60**_ **[%]**†
1‡	Mn (9)	none	0	0	0	95
2	Mn (9)	DMSO	60	0	20	trace
3	Mn (0)	DMSO	0	0	0	95
4	Mn (9)	DMF	0	0	96	0
5	Mn (9)	CH_3_CN	0	0	0	95
6	Mn (9)	THF	0	0	0	95
7	Mn (9)	EtOH	0	0	0	95
8	Mn (3)	DMSO	87 (85)	0	12	0
9	MnO_2_ (3)	DMSO	0	0	0	99
10	Mn(OAc)_3_•2H_2_O (3)	DMSO	0	0	0	99
11	MnCl_2_•4H_2_O (3)	DMSO	0	0	0	99
12‖	Zn (3)	DMSO	55	15	11	0
13	Fe (3)	DMSO	0	54	0	44
14	Mg (3)	DMSO	0	0	0	99
15	Cu (3)	DMSO	0	0	0	99
16	Pd/C (3)	DMSO	0	0	0	99
17	Raney Ni (3)	DMSO	0	0	0	99

Reaction conditions (entries 1–7): Reaction conditions (entries 1–7): C_60_ (0.03 mmol), Mn (9 equiv), **1a** (4 equiv), ODCB (4 mL), cosolvent (0.3 mL) under an argon atmosphere for 12 h at room temperature. Reaction conditions (entries 8–13): C_60_ (0.03 mmol), metal (3 equiv), **1a** (2.2 equiv), ODCB (4 mL), DMSO (0.3 mL) under an argon atmosphere for 47 h at room temperature.

^†^HPLC yields were determined using C_70_ as an internal standard. Isolated yield is shown in parenthesis.

^‡^Reaction time is 44 h.

^‖^The reaction time is 20 h.
