# Supplementary material for: Manganese powder promoted highly efficient and selective synthesis of fullerene mono- and biscycloadducts at room temperature
Source: Sci Rep. 2015 Sep 9;5:13920. doi: 10.1038/srep13920 (PMC4563593; doi:10.1038/srep13920)
Supplement: Supplementary Information [file srep13920-s1.pdf]

Supplementary Information

Manganese powder promoted highly efficient and selective synthesis of fullerene mono- and biscycloadducts at room temperature

Weili Si[1], Xuan Zhang[1], Shirong Lu[1], Takeshi Yasuda[2], Naoki Asao[1], Liyuan Han[2], Yoshinori Yamamoto[1,3] and Tienan Jin[1,*]

[1] WPI-Advanced Institute for Materials Research (WPI-AIMR), Tohoku University, Sendai 980-8577, Japan

[2] Photovoltaic Materials Unit, National Institute for Materials Science, Tsukuba 305-0047, Japan.

[3] State Key Laboratory of Fine Chemicals, Dalian University of Technology, Dalian 116012, China

tjin@m.tohoku.ac.jp

General Information. ^{1}H NMR and ^{13}C NMR spectra were recorded on JEOL JMTC-270/54/SS (JASTEC, 400 MHz) spectrometers. ^{1}H NMR spectra are reported as follows: chemical shift in ppm (δ) relative to the chemical shift of $CDCl_3$ at 7.26 ppm, integration, multiplicities (s = singlet, d = doublet, t = triplet, q = quartet, m = multiplet and br = broadened), and coupling constants (Hz). ^{13}C NMR spectra reported in ppm (δ) relative to the central line of triplet for $CDCl_3$ at 77 ppm. High-resolution mass spectra were obtained on a BRUKER APEXIII spectrometer. Preparative recycling HPLC was used a LC-2000 Plus instrument equipped with a Buckyprep column (4.6 mm x 250 mm, nakarai Tesque). HPLC analysis performed using toluene as an elution at 0.6 mL/min flow rate, detection at 320 nm in 16 ^{o}C. Column chromatography was carried out employing Slica gel 60 N (spherical, neutral, 40~100 m, KANTO Chemical Co.). Analytical thin-layer chromatography (TLC) was performed on 0.2 mm precoated plate Kieselgel 60 F_{254} (Merk).

Materials. Anhydrous 1,2-dichlorobenzene (Aldrich), DMSO, toluene, carbon disulfide (WAKO), Mn (Mitsuwa's Pure Chem, 200mesh), C_{60} and C_{70} (Aldrich), dibromides (Aldrich) were purchased and used as received. Other dibromides were prepared through bromination by NBS.[1] The structures of products were determined by using ^{1}H NMR, ^{13}C NMR, and HMRS. The known compounds were confirmed by comparison with reported literatures.[1a,2]

[1] a) S. Lu, W. Si, M. Bao, Y. Yamamoto, T. Jin, *Org. Lett.* **2013**, *15*, 4030; b) G. P. Maller, J. Mack, *Org. Lett.* **2000**, *2*, 3979.

[2] J. C.Hummelen, B. W. Knight, F. LePeq, F. Wudl, *J. Org. Chem*. **1995**, *60*, 532.

Figure S1. Procedure for synthesis of methyl 5,5-dibromo-5-phenylpentanoate (1k):

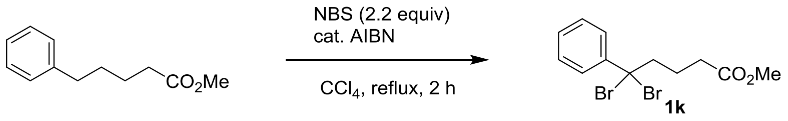

N-Bromosuccinimide (1.95 g, 11 mmol) and trace amount of azobisisobutyronitrile (AIBN) were added to a solution of methyl 5-phenylpentanoate (0.95 ml, 5 mmol) in 50 ml of carbon tetrachloride under an argon atmosphere. The mixture was heated at reflux for 2 h. The mixture was cooled to room temperature and filtered. The filtrate was removed under the reduced pressure. Upon recrystallization of the resulting residue using ethyl acetate and hexane, the desired methyl 5,5-dibromo-5-phenylpentanoate (**1k**) was obtained in 90% yield (1.6 g). White solid; ^{1}H NMR (400 MHz, $CDCl_3$) δ 1.92-2.00 (2H, m), 2.43 (2H, t, *J* = 7.2 Hz), 2.87-2.91 (2H, m), 3.69 (3H, s), 7.30 (1H, dd, *J* = 7.6, 8.0 Hz), 7.36 (2H, dd, *J* = 7.6, 8.0 Hz), 7.79 (2H, d, *J* = 8.4 Hz); ^{13}C NMR (100 MHz, $CDCl_3$) δ 23.64, 32.92, 50.99, 51.73, 70.73, 126.81, 128.19, 128.99, 144.10, 173.16. HRMS (ESI) $C_{12}H_{14}Br_2O_2$ $[M+Na]^+$: 372.9232, found: 372.9232.

Figure S2. The representative procedure for synthesis of biscycloadduct *o*-QDMC (2a).

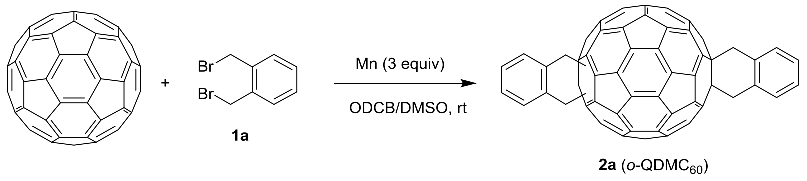

To a mixture of 1,2-dichlorobenzene (4 mL), DMSO (0.3 mL), C_{60} (21.6 mg, 0.03 mmol), and Mn (5.0 mg, 0.09 mmol, 3 equiv) was added methyl 1,2-bis(bromomethyl)benzene (**1a**, 17.42 mg, 0.066 mmol, 2.2 equiv) under an argon atmosphere. The reaction mixture was stirred at room temperature for 47 h to give a dark brown solution. After monitoring with HPLC, the mixture was purified directly by silica gel chromatography using hexane/CS_2 (1/1) as eluents. The product was washed with methanol and dried to afford the corresponding *o*-QDMC (**2a**) in 85% yield (23.6 mg).

Figure S3. The scale-up reaction of C60 with 1,3-dibromo-2,3-dihydro-1H-indene (1e) for synthesis of IC$_{60}$BA (2e)

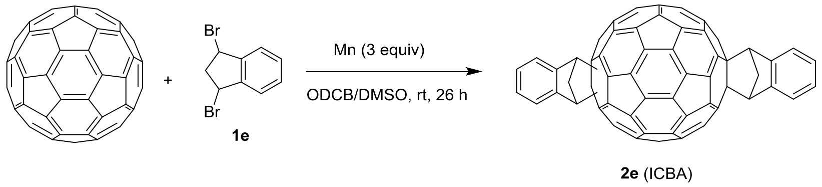

To a mixture of 1,2-dichlorobenzene (20 mL), DMSO (1.5 mL), C_{60} (108 mg, 0.15 mmol), and Mn (24.7 mg, 0.45 mmol, 3 equiv.) was added methyl 1,3-dibromo-2,3-dihydro-1H-indene (**1e**, 91 mg, 0.33 mmol, 2.2 equiv.) under an argon atmosphere. The reaction mixture was stirred at room temperature for 26 h to give a dark brown solution. After monitoring with HPLC, the mixture was purified directly by silica gel chromatography using hexane/CS_2 (1/1) as eluents. The product was washed with methanol and dried to afford the corresponding IC$_{60}$BA (**2e**) in 72% yield (103 mg).

Figure S4. The scale-up reaction of C60 with 1,3-dibromo-1,3-diphenylpropane (1f) for synthesis of 2e

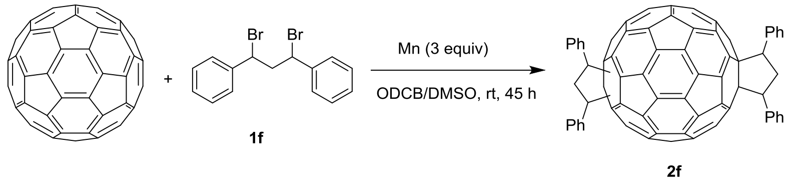

To a mixture of 1,2-dichlorobenzene (20 mL), DMSO (1.5 mL), C_{60} (108 mg, 0.15 mmol), and Mn (24.7 mg, 0.45 mmol, 3 equiv.) was added methyl 1,3-dibromo-1,3-diphenylpropane (**1f**, 117 mg, 0.33 mmol, 2.2 equiv.) under an argon atmosphere. The reaction mixture was stirred at room temperature for 45 h to give a dark brown solution. After monitoring with HPLC, the mixture was purified directly by silica gel chromatography using hexane/CS_2 (1/1) as eluents. The product was washed with methanol and dried to afford the corresponding biscycloadduct **2f** in 75% yield (125 mg).

Figure S5. The procedure for synthesis of bisPC$_{61}$BM (2k).

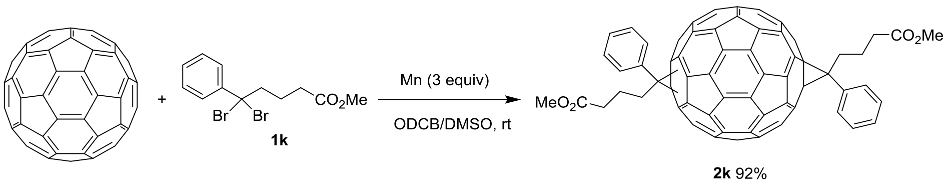

To a mixture of 1,2-dichlorobenzene (4 mL), DMSO (0.3 mL), C_{60} (21.6 mg, 0.03 mmol), and Mn (5.0 mg, 0.09 mmol, 3 equiv) was added methyl 5,5-dibromo-5-phenylpentanoate (**1k**, 23.1 mg, 0.066 mmol, 2.2 equiv) under an argon atmosphere. The reaction mixture was stirred at room temperature for 32 h to give a dark brown solution. After monitoring with HPLC, the mixture was purified directly by silica gel chromatography using toluene as an eluent. The product was washed with affording the corresponding bisPC$_{61}$BM (**2k**) in 92% yield (30.4 mg).

Figure S6. The procedure for the synthesis of PC$_{61}$BM (3g).

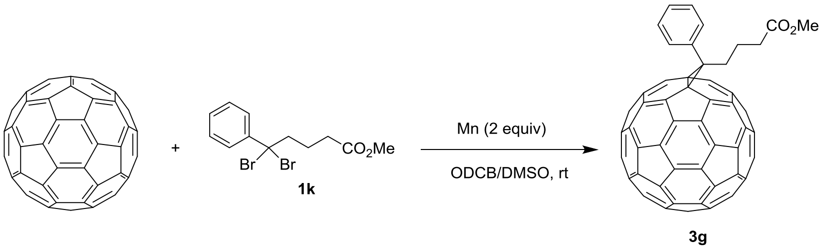

To a mixture of 1,2-dichlorobenzene (4 mL), DMSO (0.4 mL), C_{60} (21.6 mg, 0.03 mmol), and Mn (3.3 mg, 0.06 mmol, 2 equiv) was added methyl 5,5-dibromo-5-phenylpentanoate (**1k**, 10.5 mg, 0.03 mmol, 1 equiv) under an argon atmosphere in glove box. The reaction mixture was stirred at room temperature for 7 h to give a dark brown solution. After monitoring with HPLC, the mixture was purified directly by silica gel column chromatography using toluene as an eluent. The isolated product was washed with methanol and dried to afford PC$_{61}$BM (**3g**) in 93% yield (25.3 mg).

The large scale reaction of C60 with methyl 5,5-dibromo-5-phenylpentanoate (1k) for the synthesis of PC$_{61}$BM (3g).

To a mixture of 1,2-dichlorobenzene (40 mL), DMSO (4 mL), C_{60} (200 mg, 0.277 mmol), and Mn (30.4 mg, 0.554 mmol, 2 equiv) was added methyl 5,5-dibromo-5-phenylpentanoate (**1k**, 97 mg, 0.277 mmol, 1 equiv) under an argon atmosphere in glove box. The reaction mixture was stirred at room temperature for 12 h to give a dark brown solution. After monitoring with HPLC, the mixture was purified directly by silica gel column chromatography using toluene as an eluent. The isolated product was washed with methanol and dried to afford PC$_{61}$BM (**3g**) in 88% yield (222 mg).

Figure S7. Synthesis of PC$_{61}$BM under the Co-catalyzed cyclization method (our previously reported method)[1a]

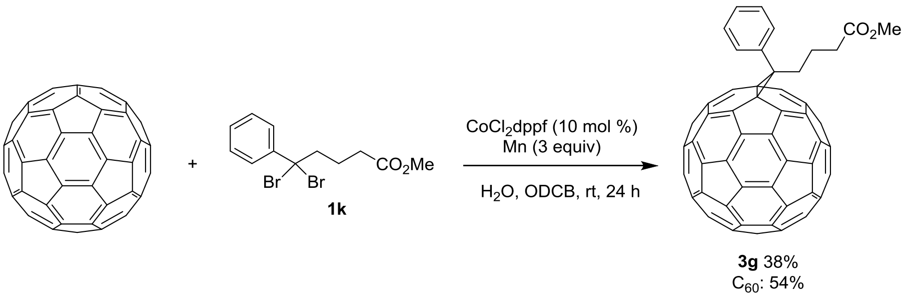

Figure S8. The procedure for the synthesis of PC$_{71}$BM (3h).

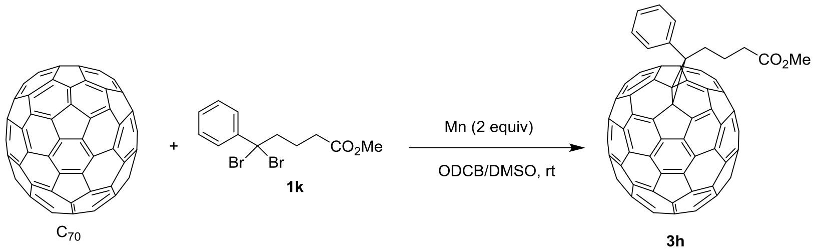

To a mixture of 1,2-dichlorobenzene (4 ml), DMSO (0.4 mL), C_{70} (25.2 mg, 0.03 mmol), and Mn (3.3 mg, 0.06 mmol, 2 equiv) was added methyl 5,5-dibromo-5-phenylpentanoate (**1k**, 10.5 mg, 0.03 mmol, 1 equiv) under an argon atmosphere in glove box. The reaction mixture was stirred at room temperature for 12 h to give a dark brown solution. After monitoring with HPLC, the mixture was purified directly by silica gel column chromatography using toluene as an eluent. The resulting mixture of [6,6]- and [5,6]-isomers was further heated at 180 °C for 24 h. After concentration, the residue was washed with methanol and dried to afford the corresponding [6,6]-isomers PC$_{71}$BM (**3h**) in 90% yield (28 mg).

Analytic data and crude mixture of HPLC spectra (C70 was used as an internal standard)

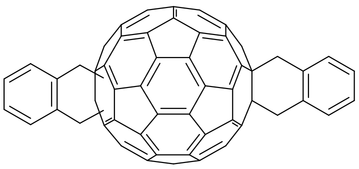

2a (*o*-QDMC$_{60}$)

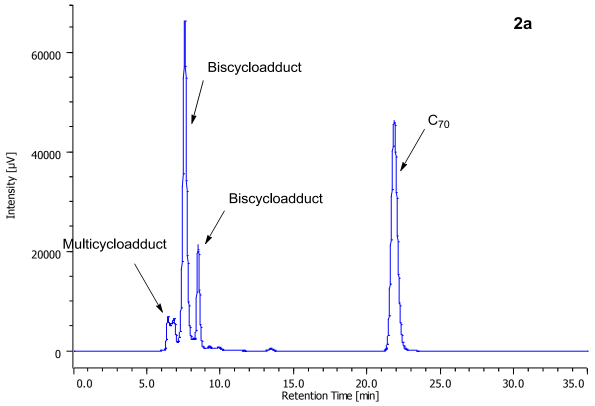

2a: dark brown solid; ^{1}H NMR (400 MHz, $CDCl_3$) δ 3.49-5.00(8H, m), 7.37-7.83 (8H, m); ^{13}C NMR (100

MHz, $CDCl_3/CS_2$ = 1/4) δ 43.52, 44.11, 44.46, 44.79, 45.22, 63.93, 64.05, 64.34, 64.42, 64.65, 127.26, 127.51, 127.69, 137.05, 137.18, 137.45, 140.88, 141.15, 142.38, 142.58, 143.31, 143.99, 144.13, 144.31, 144.44, 144.75, 144.93, 144.99, 145.52, 145.91, 146.26, 146.38, 146.73,147.28, 147.66, 148.17, 148.79, 149.14, 154.32, 154.74, 159.80, 160.94. HRMS (MALDI) $C_{76}H_{16}$ $[M]^+$: 928.1247, found 928.1247.

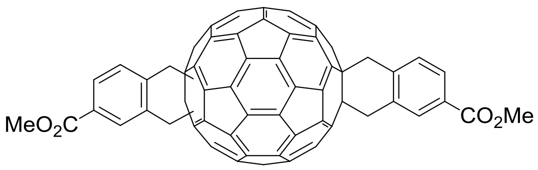

2b 70% (47 h)

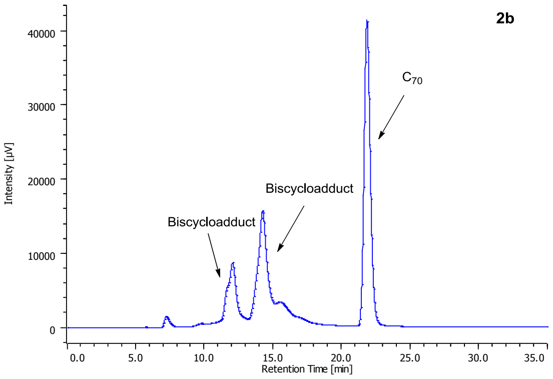

2b: Dark brown solid; ^{1}H NMR (400 MHz, $CDCl_3/CS_2$ = 1/4) δ 3.94-4.13 (8H, m), 4.33-5.01 (6H, m), 7.27-8.51 (6H, m); ^{13}C NMR (100 MHz, $CDCl_3/CS_2$ = 1/4) δ 44.28, 44.65, 45.04, 51.76, 51.81, 63.73, 63.79, 63.86, 63.92, 64.10, 64.18, 64.43, 64.50, 127.46, 127.73, 128.77, 128.98, 129.57, 137.82, 139.40, 141.00, 141.11, 141.56, 142.38, 142.78, 143.42, 144.23, 144.43, 145.01, 145.60, 145.95, 146.33, 147.72, 148.23, 149.14, 154.01, 154.35, 156.74, 159.41, 160.24,166.01; HRMS (MALDI) calcd for $C_{80}H_{20}O_4$ $[M]^+$: 1044.1356, found 1044.1358.

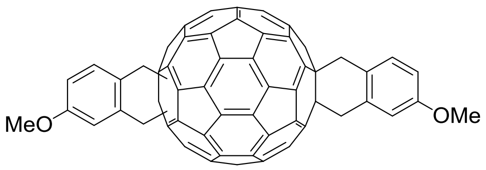

2c 71% (38 h)

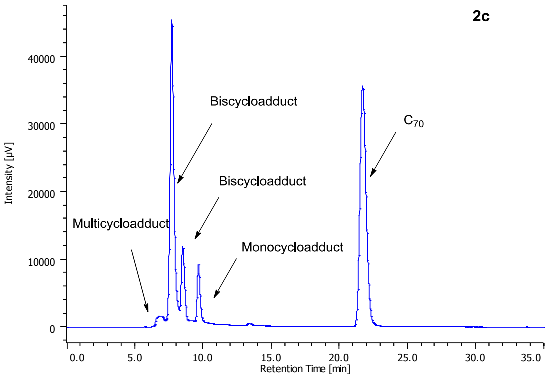

2c: Dark brown solid; ^{1}H NMR (400 MHz, $CDCl_3/CS_2$ = 1/4) δ 3.72-4.91 (14H, m), 6.87-7.66 (6H, m); ^{13}C NMR (100 MHz, $CDCl_3/CS_2$ = 1/4) δ 43.76, 44.09, 44.47, 44.79, 45.12, 45.50, 54.67, 54.70, 54.72, 63.84, 63.95, 63.97, 64.20, 64.23, 64.28, 64.34, 64.56, 64.61, 64.70, 64.92, 112.59, 113.29, 128.13, 128.40, 128.98, 129.40, 138.16, 138.55, 140.85, 141.05, 142.55, 143.29, 143.31, 144.10, 144.29, 144.45, 144.74, 144.98, 145.50, 145.83, 146.24, 146.25, 146.27, 146.35, 147.65, 148.16, 148.71, 149.04, 154.27, 154.29, 154.74, 158.82, 159.02, 159.82; HRMS (MALDI) calcd for $C_{88}H_{20}O_2$ $[M]^+$: 1108.1458, found

1108.1463.

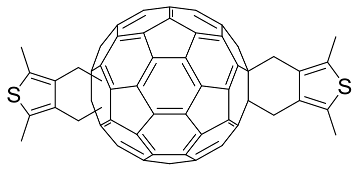

2d 56% (15 h)

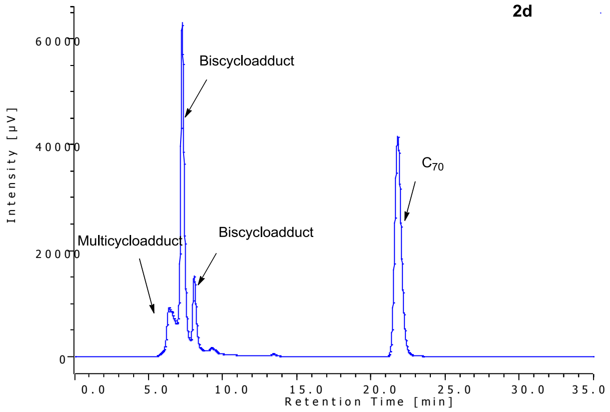

2d: Dark brown solid; ^{1}H NMR (400 MHz, $CDCl_3/CS_2$ = 1/4) δ 2.18 -2.67 (12H, m), 3.85 -4.51 (8H, m); ^{13}C NMR (100 MHz, $CDCl_3/CS_2$ = 1/4) δ 12.32, 12.47, 12.53, 12.69, 38.94, 39.54, 39.85, 40.09, 40.27, 62.67, 63.87, 64.15, 64.31, 64.51, 129.10, 129.18, 129.47, 132.59, 132.71, 132.82, 132.98, 139.38, 140.90, 141.12, 141.34, 141.98, 142.57, 143.34, 144.02, 144.16, 144.28, 144.33, 144.52, 144.77, 145.01, 145.07, 145.36, 145.52, 145.94, 146.41, 146.75, 146.80, 147.31, 147.71, 148.19, 148.74, 148.84, 149.16, 149.98, 154.12, 155.04, 160.08, 161.17. HRMS (MALDI) $C_{76}H_{20}S_2$ $[M]^+$: 996.1001, found: 996.1002.

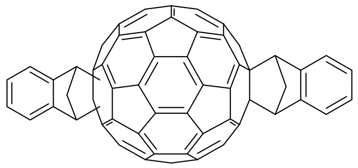

2e 75% (23 h)

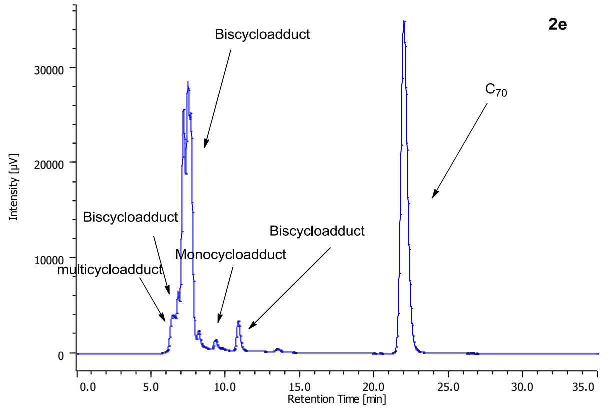

2e: Dark brown solid; ^{1}H NMR (400 MHz, $CDCl_3/CS_2$ = 1/4) δ 2.45-4.05 (4H, m), 4.36-5.19 (4H, , m), 7.09-7.82 (8H, m); ^{13}C NMR (100 MHz, $CDCl_3/CS_2$ = 1/4) δ 45.18, 45.24, 45.80, 45.91, 45.99, 46.12, 46.31, 47.17, 56.99, 57.09, 57.13, 57.48, 57.51, 57.71, 57.74, 57.82, 57.90, 57.94, 58.06, 58.13, 58.15, 58.78, 73.32, 73.48, 73.54, 73.68, 73.77, 73.88, 74.10, 74.16, 74.25, 123.08, 123.10, 123.21, 123.26, 123.33, 123.43, 123.49, 123.62, 123.65, 123.69, 123.84, 123.96, 126.50, 126.64, 126.79, 126.87, 129.93, 127.02, 127.05, 127.09, 127.22, 127.25, 135.97, 136.12, 136.21, 136.28, 136.35, 136.40, 136.50, 136.54, 137.09, 137.12, 137.34, 140.63, 140.64, 140.70, 140.81, 140.84, 140.91, 141.50, 141.56, 141.71, 141.78, 141.86, 141.96, 142.01, 142.21, 143.21, 143.43, 143.57, 144.02, 144.10, 144.15, 144.17, 144.19, 144.31, 144.36, 144.41, 144.48, 144.51, 144.59, 144.64, 144.68, 144.72, 144.76, 144.84, 144.88, 145.15, 145.19,

145.27, 145.37, 145.39, 145.45, 145.49, 145.51, 145.66, 145.82, 146.14, 146.30, 146.56, 147.10, 147.12, 147.22, 147.26, 147.39, 147.54, 147.58, 147.76, 147.96, 148.04, 148.25, 148.33, 148.49, 154.21, 154.98, 155.56, 156.29, 156.47, 157.46, 158.77; HRMS (MALDI) $C_{78}H_{16}$ $[M]^+$: 952.1247, found: 952.1246.

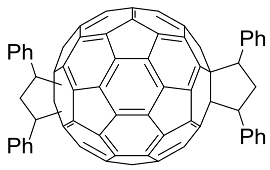

2f 77% (12 h)

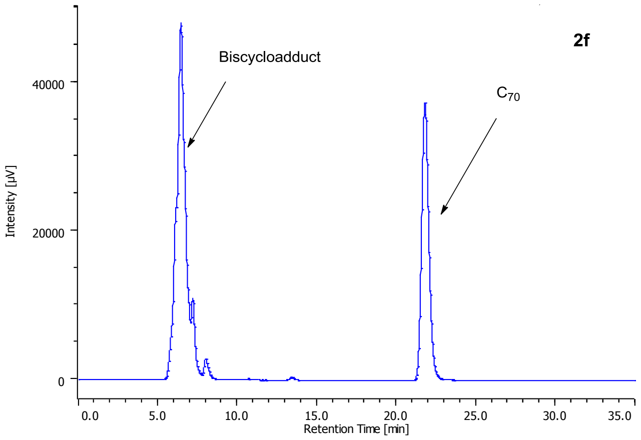

2f: Dark brown solid; soluble solvents: $CHCl_3$, toluene, ODCB; ^{1}H NMR (400 MHz, $CDCl_3/CS_2$ = 1/4) δ 2.62-5.69 (8H, m), 7.00-8.06 (20H, m); ^{13}C NMR (100 MHz, $CDCl_3/CS_2$ = 1/4) δ 33.23, 34.13, 34.73, 34.89, 34.98, 35.20, 35.24, 35.28, 35.40, 36.71, 37.21, 37.34, 37.40, 57.00, 57.14, 57.29, 57.43, 57.58, 57.73, 57.99, 58.22, 58.31, 58.70, 58.82, 58.89, 58.95, 58.99, 59.07, 59.12, 59.18, 59.26, 59.43, 59.46, 59.54, 59.66, 59.69, 59.74, 59.80, 60.32, 74.01, 74.06, 74.15, 74.21, 74.27, 74.34, 74.40, 74.42, 74.44, 74.49, 74.53, 74.55, 74.59, 74.64, 74.77, 74.82, 74.86, 74.90, 75.11, 135.08, 137.33, 137.35, 137.47, 137.50, 137.65, 137.67, 137.73, 137.83, 137.89, 138.00, 138.22, 140.34, 140.45, 140.64, 140.87, 140.90, 140.97, 141.01, 141.07, 141.15, 142.13, 143.03, 143.71, 143.75, 144.05, 144.10, 144.12, 144.18, 144.25, 144.46, 144.56, 144.62, 144.73, 144.74, 144.82, 144.85, 145.31, 145.33, 145.48, 146.02, 146.48, 146.60, 147.19, 147.38, 147.47, 147.53, 147.62, 147.66, 147.76, 148.09, 148.21, 148.26, 148.33, 148.36, 148.43, 148.50, 148.54, 148.59, 148.61, 148.67, 152.17, 152.41, 153.99, 154.17, 154.28, 155.32, 155.42, 155.82, 155.96, 157.47. HRMS (MALDI) $C_{90}H_{28}$ $[M]^+$:1108.2186, found 1108.2187.

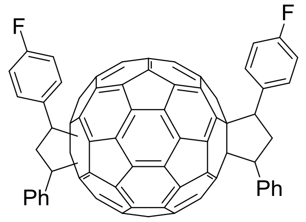

2g 81% (17 h)

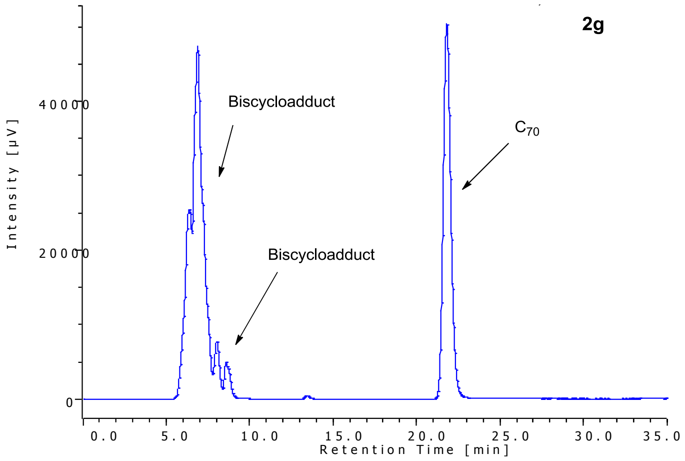

2g: Dark brown solid; soluble solvents: $CHCl_3$, toluene, ODCB; ^1H NMR (400 MHz, $CDCl_3/CS_2$ = 1/4) δ 2.41-5.72 (8H, m), 6.83-8.01 (18H, m); ^{13}C NMR (100 MHz, $CDCl_3/CS_2$ = 1/4) δ 34.97, 5.13, 35.38, 35.46, 36.81, 37.35, 56.55, 56.62, 56.71, 57.19, 57.33, 57.45, 57.58, 57.62, 57.78, 58.10, 58.53, 58.82, 58.88, 59.08, 59.40, 59.59, 59.71, 73.82, 74.11, 74.14, 74.20, 74.29, 74.34, 74.43, 74.65, 74.86, 74.94, 114.47, 144.59, 114.68, 114.80, 114.87, 114.92, 115.02, 115.08, 115.15, 115.24, 115.30, 115.36, 115.43, 115.52, 127.00, 127.06, 127.09, 127.17, 127.25, 127.32, 127.41, 127.48, 127.58, 127.90, 127.94, 127.97, 128.04, 128.07, 128.13, 128.16, 128.20, 128.24, 128.26, 128.32, 128.37, 128.45, 128.53, 128.55, 128.58, 128.72, 128.76, 128.78, 128.81, 128.85, 128.86, 128.89, 128.93, 129.02, 129.05, 129.12, 129.24, 130.03, 130.11, 130.17, 130.23, 130.30, 130.34, 130.42, 130.52, 130.57, 130.60, 130.64, 130.71, 137.52, 140.89, 140.93, 143.84, 144.12, 144.53, 144.58, 144.66, 144.70, 145.28, 147.36, 147.57, 152.38, 153.58, 153.65, 154.17, 155.01, 155.11, 157.07, 157.26, 159.29, 160.33, 160.46, 160.53, 162.74, 162.99. HRMS (MALDI) $C_{90}H_{26}F_2$ $[M]^+$: 1144.1997, found 1144.1997.

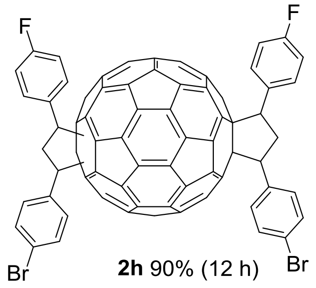

2h 90% (12 h)

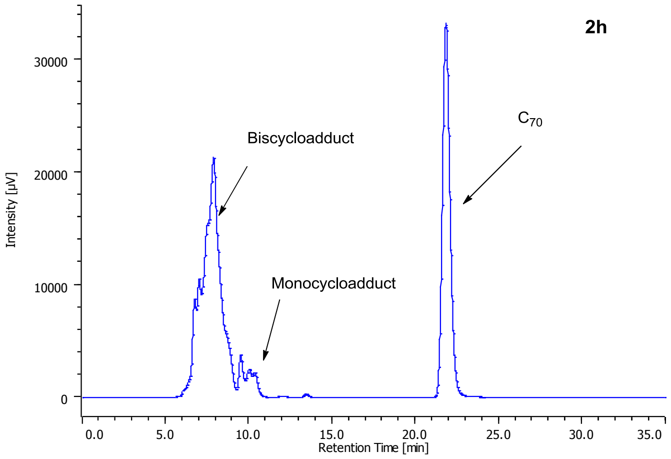

2h: Dark brown solid; ^1H NMR (400 MHz, $CDCl_3$) δ 2.38-5.68 (8H, m), 6.90-7.88 (16H, m); ^{13}C NMR (100 MHz, $CDCl_3/CS_2$=1/4) δ 33.34, 34.66, 34.84, 35.04, 35.41, 35.46, 36.43, 36.67, 56.43, 56.60, 56.70,

56.89, 56.98, 57.38, 57.46, 58.03, 58.07, 58.15, 58.21, 58.34, 58.41, 58.50, 58.78, 58.89, 59.04, 59.10, 73.61, 73.77, 73.82, 73.85, 74.00, 74.13, 74.28, 74.44, 74.59, 74.67, 114.29, 114.74, 114.92, 114.96, 115.16, 115.20, 115.30, 115.37, 115.51, 115.58, 121.53, 121.79, 121.84, 121.98, 122.01, 122.05, 122.13, 122.26,129.87, 129.91, 129.95, 130.02, 130.04, 130.09, 130.22, 130.26, 130.29, 130.34, 130.39, 130.42, 130.51, 130.56, 130.61, 130.69, 130.74, 130.80, 131.04, 131.15, 131.28, 131.36, 131.46, 131.49, 131.56, 131.59, 131.69, 136.45, 136.78, 139.00, 140.60, 140.89, 141.02, 141.26, 141.70, 141.99, 143.11, 143.31, 143.66, 143.81, 143.85, 144.07, 144.19, 144.26, 144.51, 144.56, 144.58, 144.60, 144.69, 144.71, 144.74, 144.82, 145.29, 145.84, 146.38, 146.42, 146.49, 146.61, 147.11, 147.39, 147.66, 147.71, 147.83, 148.33, 148.41, 148.49, 148.70. HRMS (MALDI) $C_{90}H_{24}Br_2F_2$ $[M]^+$:1300.0207, found 1300.0212.

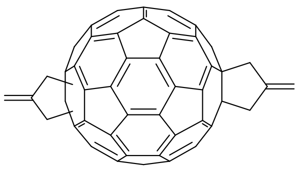

2i 61% (47 h)

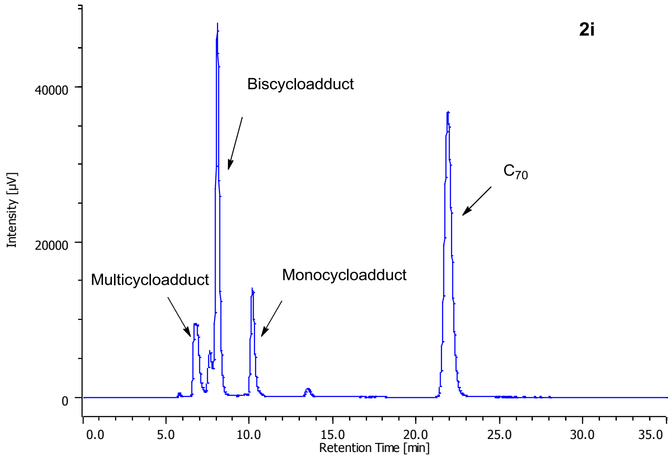

2i: Dark brown solid; ^{1}H NMR (400 MHz, $CDCl_3/CS_2$ = 1/4) δ 3.32-4.57 (8H, m), 5.19-5.69 (4H, m); ^{13}C NMR (100 MHz, $CDCl_3/CS_2$ = 1/4) δ 47.56, 47.64, 48.11, 48.37, 48.56, 67.83, 67.88, 68.07, 68.24, 68.29, 109.55, 110.06, 134.47, 134.52, 135.45, 136.32, 137.59, 139.46, 139.80, 140.18, 140.74, 140.79, 140.85, 141.06, 141.16, 141.18, 142.20, 142.82, 143.19, 143.25, 143.37, 143.55, 143.70, 144.04, 144.16, 144.22, 144.35, 144.37, 144.42, 144.65, 144.72, 144.87, 144.95, 144.97, 145.05, 145.35, 145.69, 145.75, 146.36, 146.87, 147.43, 147.67, 147.82, 147.91, 148.04, 148.26, 148.53, 148.56, 148.74, 153.87, 154.37, 154.81, 156.18, 156.94, 156.95, 159.78, 160.36. HRMS (MALDI) $C_{68}H_{12}$ $[M]^+$: 828.0934, found 828.0934.

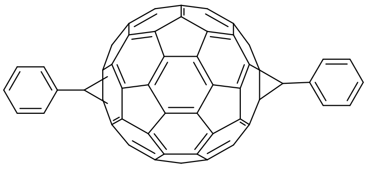

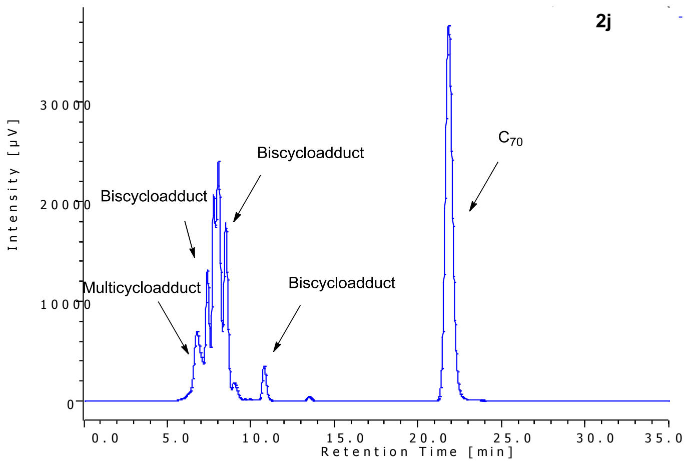

2j 90% (18 h)

2j: Dark brown solid; soluble solvents: $CHCl_3$, toluene, ODCB; ^1H NMR (400 MHz, $CDCl_3/CS_2$ = 1/4) δ 4.54-5.50 (2H, m), 7.26-8.19 (10H, m); ^{13}C NMR (100 MHz, $CDCl_3/CS_2$ = 1/4) δ 40.88, 40.94, 41.03, 41.07, 41.23, 41.90, 42.35, 42.42, 42.55, 44.56, 45.12, 70.57, 73.02, 74.29, 74.38, 74.43, 74.59, 74.62, 74.67, 75.01, 75.12, 75.14, 75.25, 75.74, 75.78, 75.86, 75.90, 127.83, 127.87, 127.90, 127.92, 127.97, 128.02, 128.04, 128.14, 128.24, 128.33, 128.38, 128.41, 128.44, 128.60, 130.27, 130.50, 130.60, 130.63, 130.73, 130.75, 130.77, 130.84, 130.87, 130.92, 131.03, 132.05, 132.43, 132.56, 132.61, 132.63, 132.67, 132.80, 132.94, 132.97, 133.07, 133.10, 137.52, 137.59, 137.87, 137.95, 138.00, 138.05, 138.20, 138.32, 138.64, 138.71, 138.76, 138.82, 139.04, 139.16, 139.27, 139.35, 139.48, 139.51, 139.76, , 140.96, 141.12, 141.21, 141.28, 141.31, 141.36, 141.51, 141.90, 141.92, 142.20, 142.55, 142.64, 142.91, 142.93, 142.95, 142.96, 143.40, 143.43, 143.52, 143.53, 143.56, 143.65, 143.74, 143.76, 143.78, 143.80, 143.83, 143.90, 143.94, 144.00, 144.02, 144.09, 144.14, 144.16, 144.25, 144.32, 144.35, 144.51, 144.68, 144.72, 144.76, 144.87, 144.98, 145.12, 145.37, 145.47, 145.64, 145.76, 145.78, 145.85, 145.88, 145.90, 145.92, 145.94, 146.02, 146.04, 146.06, 146.11, 146.19, 146.21, 147.21, 147.39, 147.93, 150.40, 151.13. HRMS (MALDI) $C_{74}H_{12}$ $[M]^+$: 900.0934, found 900.0934.

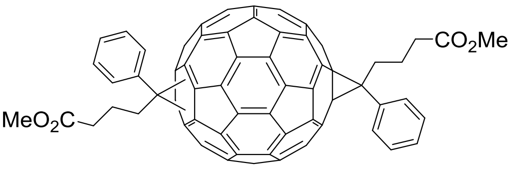

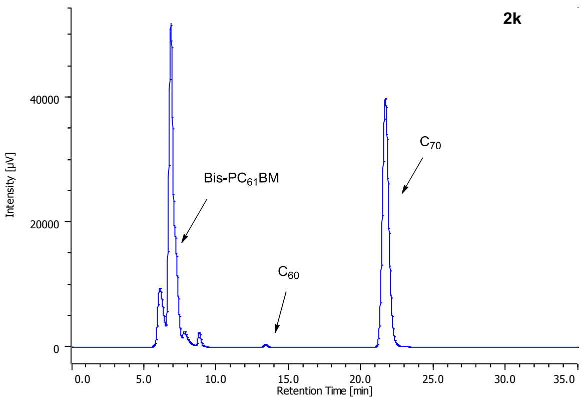

2k 92% (32 h)

2k: Dark brown solid; soluble solvents: $CHCl_3$, toluene, ODCB; ^1H NMR (400 MHz, $CDCl_3/CS_2$ = 1/4) δ 1.90-3.15 (12H, m), 3.56-3.73 (6H, m), 7.32-8.12 (10H, m); ^{13}C NMR (100 MHz, $CDCl_3/CS_2$ = 1/4) δ

22.07, 22.12, 22.18, 22.20, 22.23, 22.26, 22.29, 22.43, 22.45, 32.99, 33.38, 33.41, 33.60, 33.62, 33.64, 33.67, 33.70, 33.73, 33.84, 33.85, 34.10, 48.70, 48.79, 48.87, 48.96, 48.98, 50.37, 50.58, 50.61, 50.64, 50.67, 51.13, 51.17, 51.20, 51.22, 51.25, 51.28, 51.31, 53.15, 78.37, 78.41, 78.57, 78.61, 78.64, 78.97, 78.99, 79.49, 79.54, 79.55, 79.95, 80.20, 80.24, 127.76, 127.87, 127.99, 128.01, 128.08, 128.12, 128.17, 128.19, 128.23, 128.25, 128.30, 128.41, 131.30, 131.52, 131.70, 131.72, 131.78, 131.81, 131.83, 132.04, 132.07, 135.89, 136.19, 136.28, 136.38, 136.50, 136.56, 136.59, 136.78, 136.80, 136.82, 136.90, 136.92, 137.31, 137.34, 137.46, 137.57, 137.60, 137.68, 137.99, 138.42, 138.58, 138.89, 139.40, 139.75, 140.12, 140.15, 140.24, 140.38, 140.47, 140.52, 140.54, 140.71, 140.76, 140.80, 140.82, 140.92, 141.26, 141.31, 141.46, 141.49, 141.50, 141.52, 141.56, 141.61, 141.64, 141.70, 141.82, 142.10, 142.20, 142.34, 142.45, 142.59, 142.66, 142.77, 142.85, 142.99, 143.02, 143.03, 143.07, 143.08, 143.10, 143.13, 143.15, 143.25, 143.47, 143.50, 143.52, 143.64, 143.65, 143.77, 143.87, 143.88, 143.90, 143.97, 143.99, 144.01, 144.04, 144.06, 144.08, 144.20, 144.23, 144.25, 144.27, 144.29, 144.30, 144.33, 144.35, 144.39, 144.43, 144.53, 144.67, 144.70, 144.77, 144.96, 144.98, 144.99, 145.09, 145.12, 145.24, 145.26, 145.27, 145.33, 145.36, 145.43, 145.49, 145.63, 145.69, 145.78, 145.83, 145.89, 145.97, 146.18, 146.21, 146.26, 146.35, 146.47, 146.53, 146.69, 146.72, 146.85, 146.90, 146.96, 147.41, 147.53, 147.56, 147.61, 147.72, 148.03, 148.30, 148.35, 148.53, 148.66, 148.74, 148.93, 149.23, 149.28, 149.53, 149.60, 149.62, 150.45, 150.50, 151.37, 172.61, 172.63, 172.66, 172.69, 172.71, 172.77. HRMS (MALDI) $C_{84}H_{28}O_4$ [M]+: 1100.1982, found: 1100.1985.

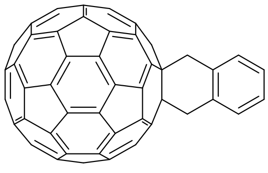

3a 72% (12 h)

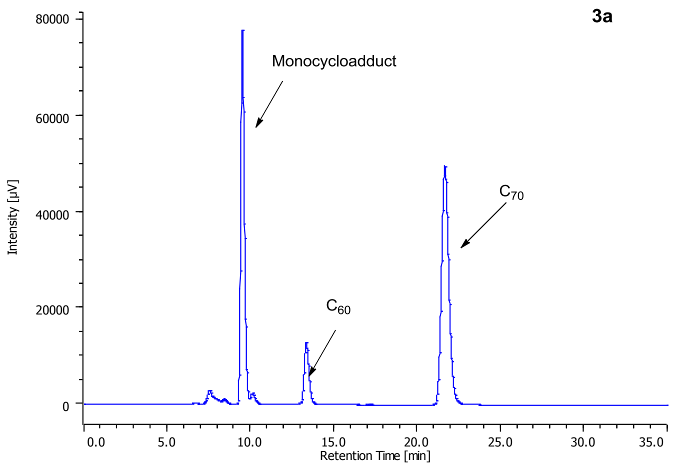

3a: Dark brown solid; soluble solvents: toluene, ODCB, CS_2, low in $CHCl_3$; ^{1}H NMR (400 MHz, $CDCl_3/CS_2$ = 1/4) δ 4.45 (2H, d, J = 13.2 Hz), 4.84 (2H, d, J = 13.2 Hz), 7.52-7.56 (2H, m), 7.63-7.67 (2H, m); ^{13}C NMR (100 MHz, $CDCl_3/CS_2$ = 1/4) δ 44.98, 65.53, 127.59, 127.83, 135.04, 135.88, 137.59, 139.78, 141.25, 141.69, 141.80, 142.21, 142.75, 144.31, 144.70, 145.05, 145.39, 145.85, 146.09, 147.25, 156.29; HRMS (MALDI) $C_{68}H_8$ [M]+: 824.0621, found: 824.0621.

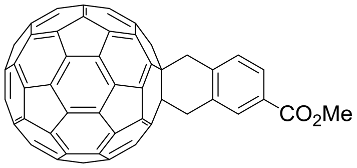

3b 82% (12 h)

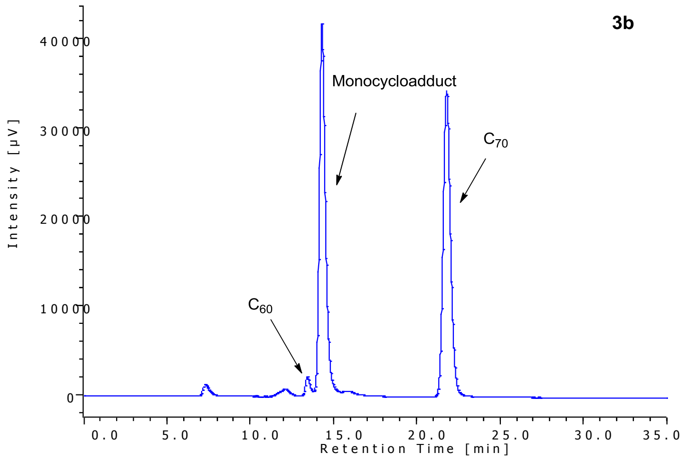

3b: Dark brown solid; soluble solvents: toluene, ODCB, CS_2, low in $CHCl_3$; ^1H NMR (400 MHz, $CDCl_3/CS_2$ = 1/4) δ 3.99 (3H, s), 4.53 (2H, d, J = 12.4 Hz), 4.88 (2H, d, J = 12.0 Hz), 7.74 (1H, d, J = 7.6 Hz), 8.22 (1H, d, J = 8.0 Hz), 8.32(1H, s); ^{13}C NMR (100 MHz, $CDCl_3/CS_2$ = 1/4) δ 44.79, 44.98, 51.58, 65.15, 65.23, 127.61, 128.73, 129.19, 129.80, 137.77, 141.31, 141.65, 142.20, 142.58, 144.22, 144.27, 145.03, 145.31, 145.83, 146.06, 146.08, 147.21, 147.24, 165.39. HRMS (MALDI) $C_{70}H_{10}O_2$ [M]+: 882.0675, found: 882.0676.

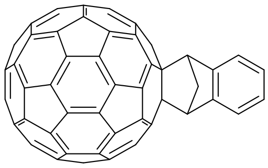

3c 74% (24 h)

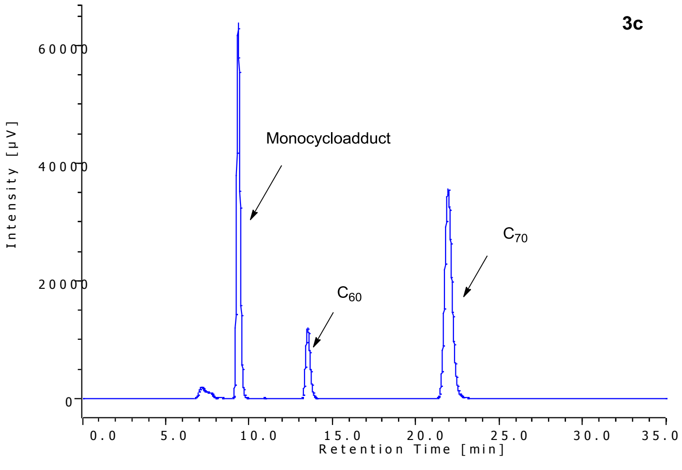

Dark brown solid; soluble solvents: toluene, ODCB, CS_2, low in $CHCl_3$; ^1H NMR (400 MHz, $CDCl_3/CS_2$ = 1/4) δ 2.94 (1H, dt, J = 10, 1.6 Hz), 3.88 (1H, dt, J = 10, 1.6 Hz), 4.94 (2H, t, J = 1.6 Hz), 7.41-7.45 (2H, m), 7.59-7.63 (2H, m); ^{13}C NMR (100 MHz, $CDCl_3/CS_2$ = 1/4) δ 46.12, 58.08, 123.63, 127.21, 136.99, 137.25, 139.30, 139.82, 141.40, 141.42, 141.58, 141.67, 141.78, 141.82, 142.11, 142.25, 142.48, 142.66, 142.99, 144.17, 144.72, 144.80, 144.96, 145.03, 145.22, 145.41, 145.55, 145.59, 145.67, 145.75, 145.88, 146.77, 154.35, 155.73. HRMS (MALDI) $C_{69}H_8$ [M]+: 836.0621, found: 836.0622.

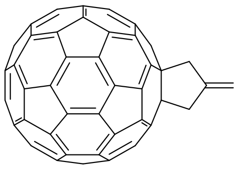

3d 60% (48 h)

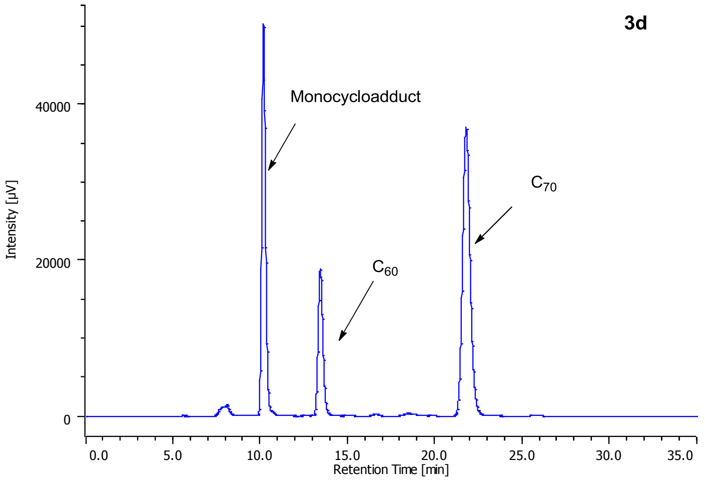

3d: Dark brown solid; soluble solvents: toluene, ODCB, CS_2, low in $CHCl_3$; ^{1}H NMR (400 MHz, $CDCl_3/CS_2$ = 1/4) δ 4.34 (4H, s), 5.66 (2H, s); ^{13}C NMR (100 MHz, $CDCl_3/CS_2$ = 1/4) δ 48.57, 69.11, 110.43, 135.20, 139.77, 141.47, 141.67, 141.87, 142.21, 142.69, 142.76, 143.56, 144.20, 144.92, 144.98, 145.22, 145.34, 145.73, 145.87, 147.00, 156.19. HRMS (MALDI) $C_{64}H_6$ [M]+: 774.0464, found: 774.0464.

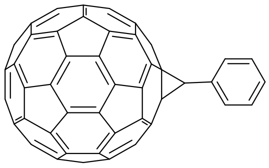

3e 81% (24 h)

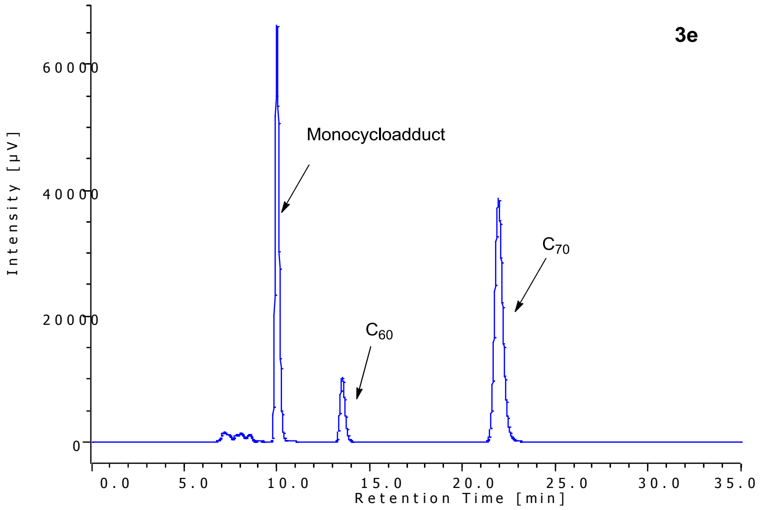

3e: Dark brown solid; soluble solvents: toluene, ODCB, CS_2, low in $CHCl_3$; ^{1}H NMR (400 MHz, $CDCl_3/CS_2$ = 1/4) δ 5.41 (1H, s), 7.44-7.49 (1H, m), 7.52-7.56 (2H, m), 7.95-7.98 (2H, m); ^{13}C NMR (100 MHz, $CDCl_3/CS_2$ = 1/4) δ 43.43, 74.95, 128.06, 128.44, 130.79, 132.48, 136.04, 138.05, 140.46, 140.69, 141.64, 141.68, 141.78, 142.23, 142.48, 142.53, 142.65, 143.24, 143.27, 143.78, 143.94, 144.09, 144.21, 144.27, 144.60, 144.65, 144.71, 144.99, 145.07, 146.91, 149.08. HRMS (MALDI) $C_{67}H_6$ [M]+: 810.0464, found: 810.0464.

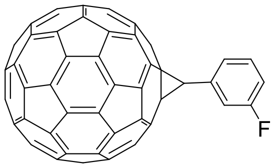

3f 75% (24 h)

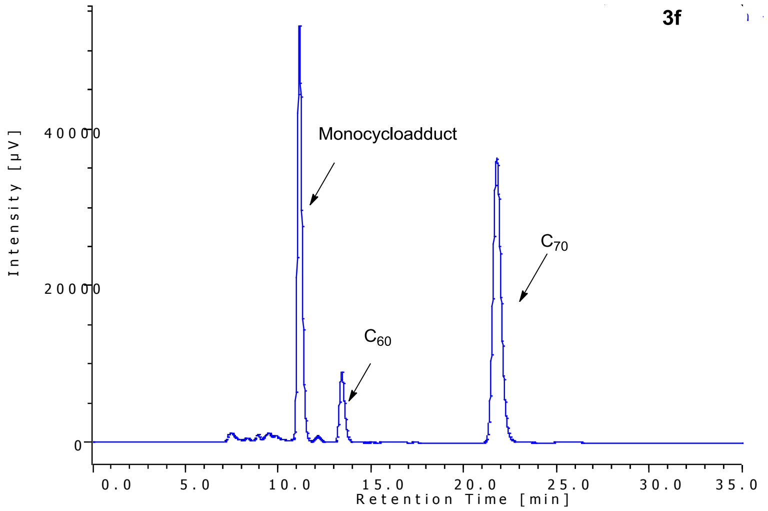

3f: Dark brown solid; soluble solvents: toluene, ODCB, CS_2, low in $CHCl_3$; ^1H NMR (400 MHz, $CDCl_3/CS_2$ = 1/4) δ 5.38 (1H, s), 7.15-7.20 (1H, m), 7.51-7.57 (1H, m), 7.66-7.68 (1H, m), 7.77-7.79 (1H, m); ^{13}C NMR (100 MHz, $CDCl_3/CS_2$ = 1/4) δ 42.57 (d, J^7 = 1.7 Hz), 74.65, 115.25 (d, J^3 = 20.6 Hz), 117.94 (d, J^2 = 21.4 Hz), 126.56 (d, J^6 = 3.3 Hz), 130.06 (d, J^4 = 8.2 Hz), 135.03 (d, J^5 = 7.4 Hz), 136.19, 138.15, 140.64, 140.83, 141.75, 141.81, 141.89, 142.30, 142.63, 142.67, 142.78, 143.35, 143.39, 143.96, 144.10, 144.16, 144.35, 144.37, 144.76, 144.81, 144.86, 145.00, 145.12, 146.60, 148.84, 162.29 (d, J^1 = 247.5 Hz); HRMS (MALDI) $C_{67}H_5F$ [M]+: 828.0370, found: 828.0369.

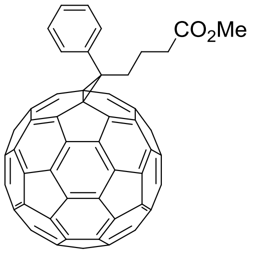

3g 93% (7 h, $PC_{61}BM$)

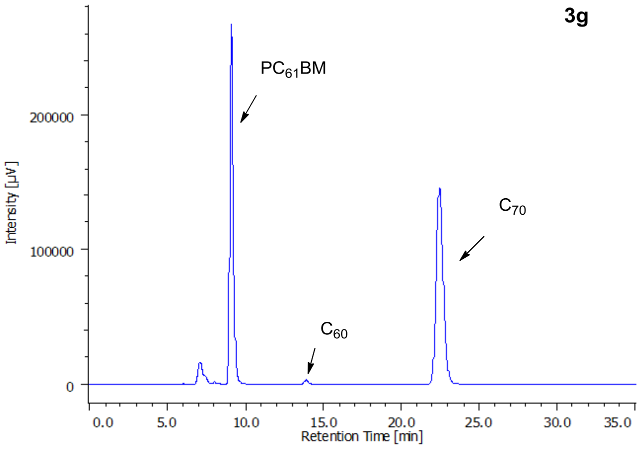

3g: Dark brown solid; soluble solvents: $CHCl_3$, toluene, ODCB; ^1H NMR (400 MHz, $CDCl_3/CS_2$ = 1/4) δ 2.15-2.23 (2H, m), 2.52 (2H, t, J = 7.2 Hz), 2.89-2.93 (2H, m), 3.67(3H, s), 7.47 (1H, dd, J = 6.8, 7.2 Hz), 7.54 (2H, dd, J = 7.2, 7.6 Hz), 7.91 (2H, d, J = 6.8 Hz); ^{13}C NMR (100 MHz, $CDCl_3/CS_2$ = 1/4) δ 22.24, 33.46, 33.57, 51.18, 51.54, 79.45, 128.01, 128.23, 131.73, 136.31, 137.34, 137.75, 140.47, 140.71, 141.78, 141.81, 141.82, 141.89, 142.62, 142.68, 142.73, 142.81, 143.44, 143.76, 144.15, 144.16, 144.34, 144.38, 144.46, 144.72, 144.74, 144.84, 144.87, 145.50, 147.37, 148.30, 172.43. HRMS (MALDI) $C_{72}H_{14}O_2$

[M]+: 910.0988, found: 910.0990.

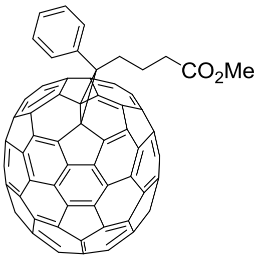

3h 90% ($PC_{71}BM$)

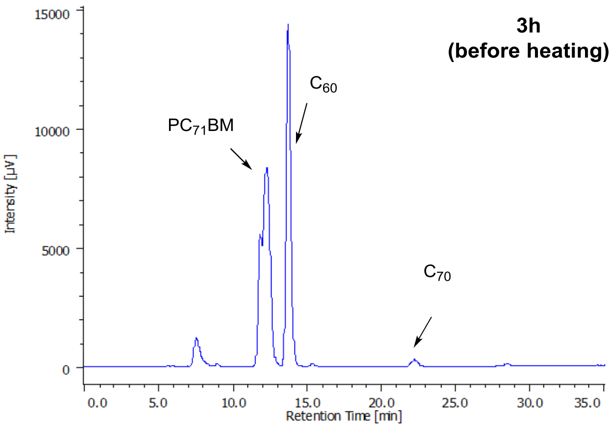

C60 was used as an internal standard.

LC-MS: before heating; a mixture of [6,6]- and [5,6]-isomers

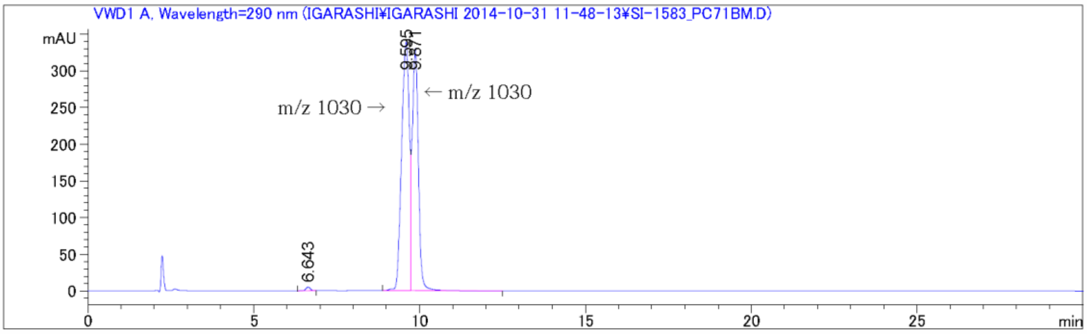

HPLC chart of **3h** after heating

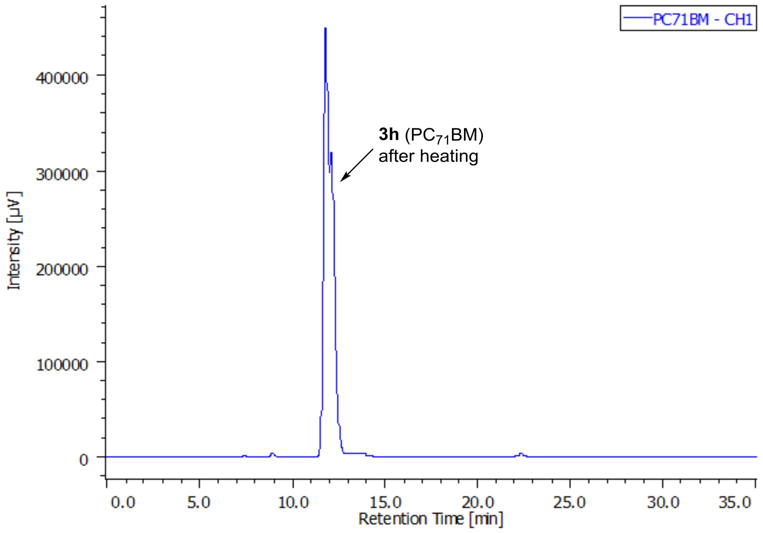

3h (after heating): ratio of 3 isomers: 38:46:16; Dark brown solid; soluble solvents: $CHCl_3$, toluene, ODCB; ^1H NMR (400 MHz, $CDCl_3/CS_2$ = 1/4) δ 1.77-2.51 (6H, m), 3.50-3.73 (3H, m), 7.20-7.88 (5H, m); ^{13}C NMR (100 MHz, $CDCl_3/CS_2$ = 1/4) δ 21.55, 21.62, 21.99, 33.22, 33.52, 33.56, 33.94, 35.09, 35.67, 37.17, 37.60, 51.03, 51.19, 51.25, 65.01, 65.28, 69.48, 71.55, 126.73, 127.54, 127.71, 128.01, 128.05, 128.07, 128.37, 128.40, 128.73, 130.18, 130.38, 130.44, 130.47, 130.61, 130.88, 130.92, 131.17, 131.29, 131.36, 131.52, 131.86, 131.88, 131.94, 132.20, 132.52, 133.53, 133.71, 136.34, 136.93, 137.56, 137.69, 138.44, 139.09, 139.35, 139.90, 140.75, 140.91, 141.08, 141.26, 141.29, 141.36, 141.45, 141.50, 142.16, 142.27, 142.38, 142.41, 142.48, 142.76, 142.81, 143.00, 143.08, 143.23, 143.36, 143.47, 143.60, 143.66, 143.82, 143.86, 143.89, 143.93, 144.20, 144.23, 144.29, 144.48, 144.51, 144.60, 145.04, 145.19, 145.22, 145.33, 145.41, 145.54, 145.56, 145.60, 145.67, 145.80, 145.95, 146.00, 146.21, 146.48, 146.52, 146.53, 146.58, 146.71, 146.77, 146.85, 146.87, 146.90, 147.08, 147.14, 147.20, 147.24, 147.49, 147.60, 147.61, 147.79, 147.88, 147.90, 148.01, 148.05, 148.11, 148.17, 148.19, 148.24, 148.26, 148.30, 148.33, 148.52, 148.81, 148.83, 148.86, 148.90, 148.94, 149.05, 149.07, 149.09, 149.12, 149.14, 149.25, 149.30, 149.34, 149.44, 149.60, 149.68, 150.20, 150.26, 150.49, 150.55, 150.80, 150.86, 151.09, 151.16, 151.67, 151.90, 152.85, 152.87, 154.91, 155.61, 172.06, 172.31, 172.35. HRMS (MALDI) $C_{82}H_{14}O_2$ [M]+: 1030.0988, found: 1030.0988.Dark brown solid; soluble solvents: $CHCl_3$, toluene, ODCB; ^1H NMR (400 MHz, $CDCl_3/CS_2$ = 1/4) δ 0.82-1.33 (2H, m), 1.77-2.21 (2H, m), 2.41-2.48 (2H, m), 3.38-3.73 (3H, m), 6.64-7.93 (5H, m); ^{13}C NMR (100 MHz, $CDCl_3/CS_2$ = 1/4) δ 19.15, 21.62, 21.70, 22.07, 28.14, 33.28, 33.32, 33.58, 33.62, 33.99, 35.14, 35.72, 37.65, 51.08, 51.24, 52.62, 65.29, 69.48, 71.55, 100.58, 125.28-155.50(m), 171.91, 172.22, 172.26. HRMS (MALDI) $C_{82}H_{14}O_2$ [M]+: 1030.0988, found: 1030.0990.

NMR spectra

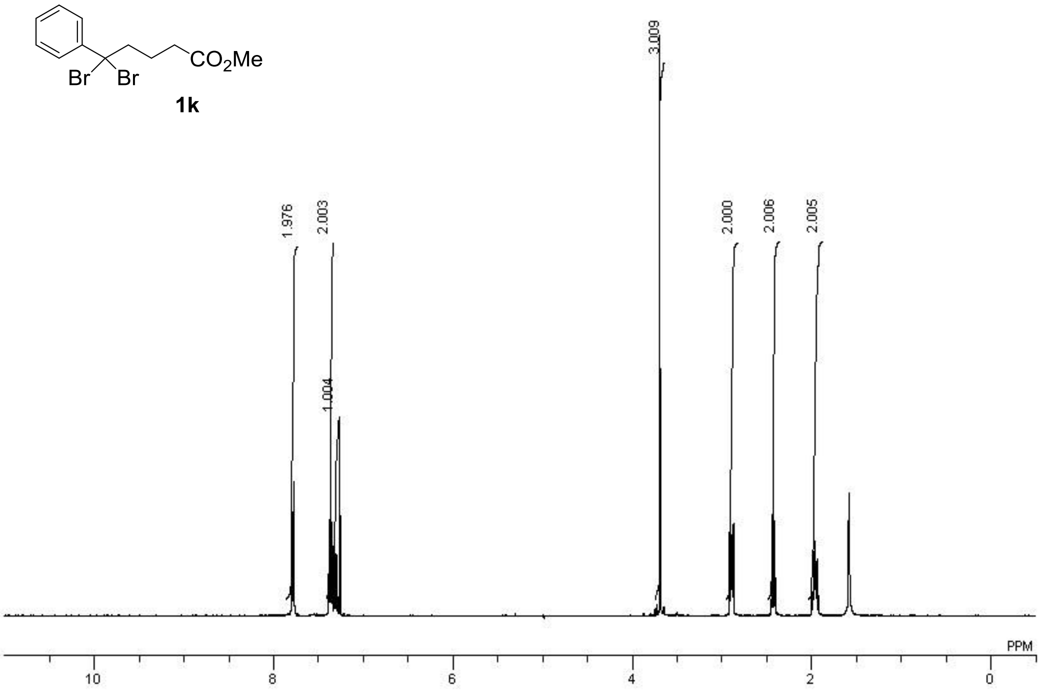

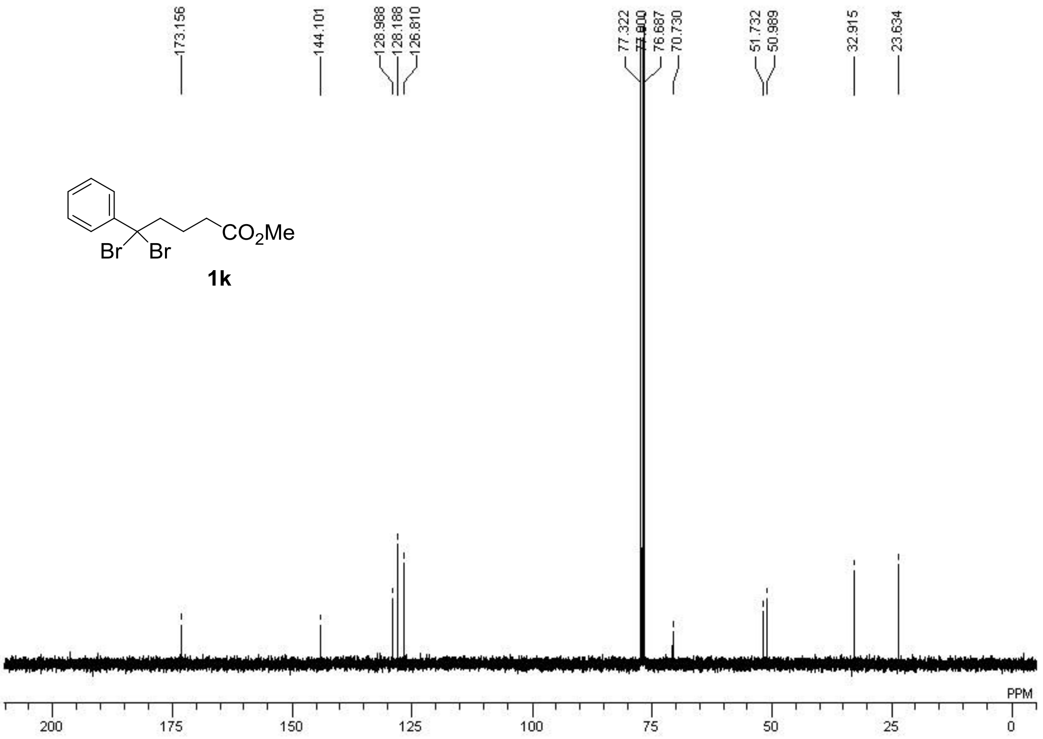

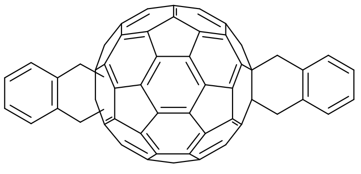

2a (o-QDMC$_{60}$)

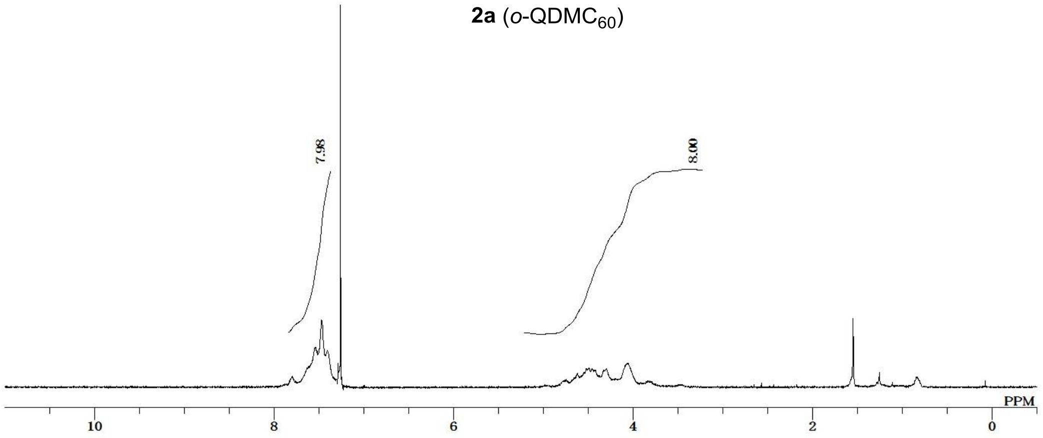

7.98
8.00
PPM
10
8
6
4
2
0

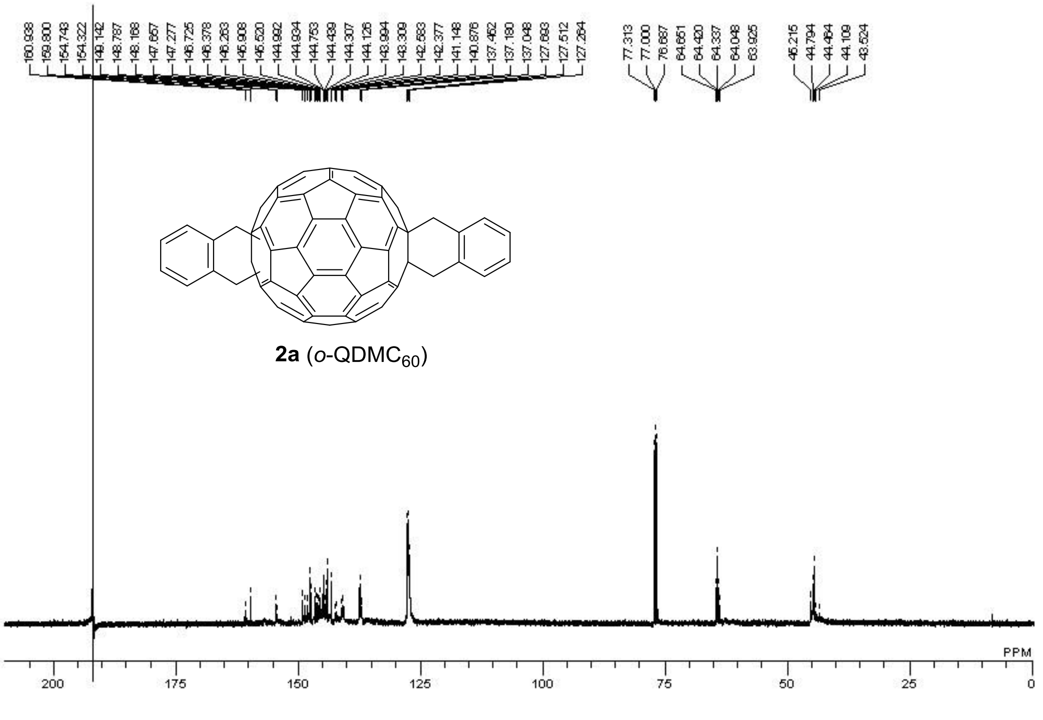

160.938
159.800
154.743
154.322
149.142
148.787
148.168
147.657
147.277
146.725
146.378
146.263
145.908
145.520
144.992
144.934
144.753
144.439
144.307
144.126
143.994
143.309
142.583
142.377
141.148
140.876
137.452
137.180
137.048
127.693
127.512
127.264
77.313
77.000
76.687
64.651
64.420
64.337
64.048
63.925
45.215
44.794
44.464
44.109
43.524
2a (o-QDMC60)
PPM
200
175
150
125
100
75
50
25
0

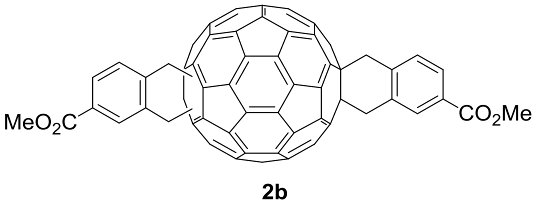

MeO2C
CO2Me

2b

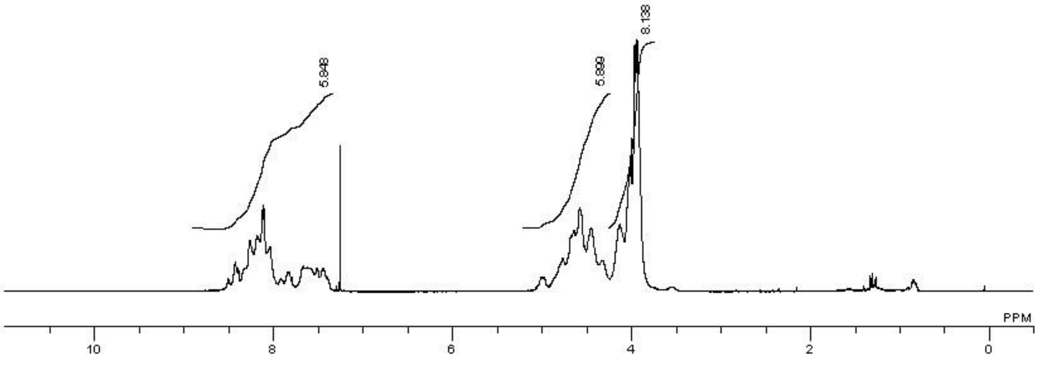

5.848
5.899
8.138
PPM
10
8
6
4
2
0

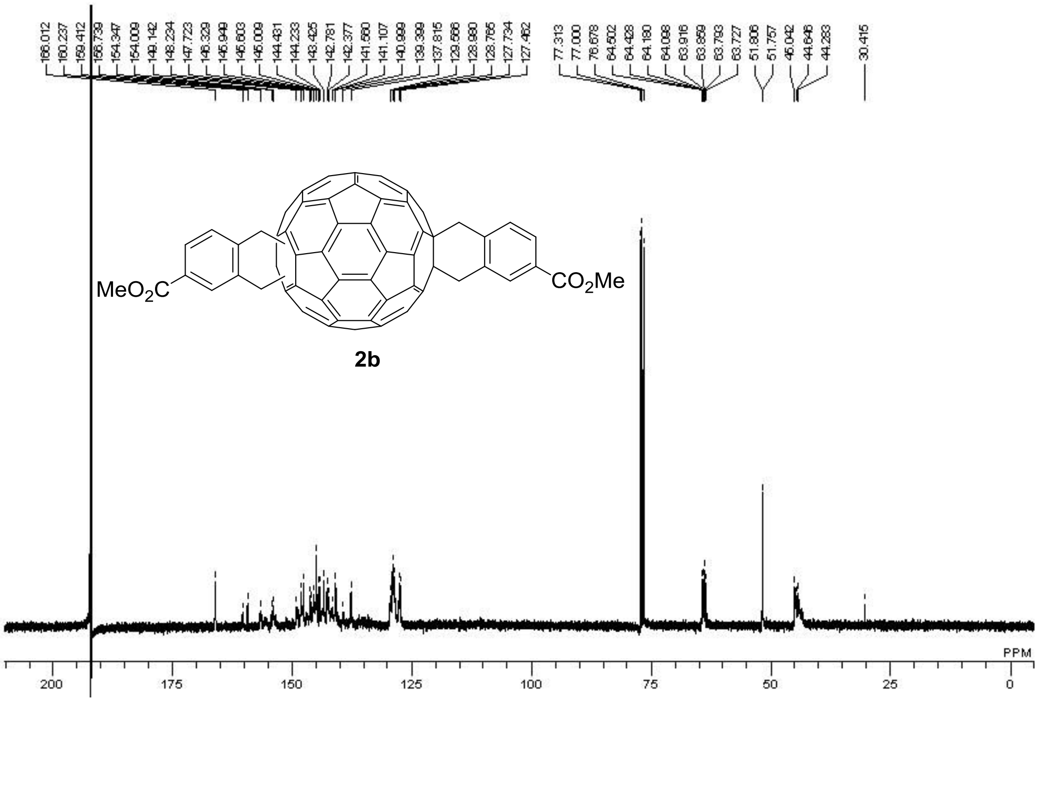

166.012
160.237
159.412
156.739
154.347
154.009
149.142
148.234
147.723
146.329
145.949
145.603
145.009
144.431
144.233
143.425
142.781
142.377
141.560
141.107
140.999
139.399
137.815
129.566
128.980
128.765
127.734
127.462
77.313
77.000
76.678
64.502
64.428
64.180
64.098
63.916
63.859
63.793
63.727
51.806
51.757
45.042
44.646
44.283
30.415
MeO2C
CO2Me
2b
PPM
200
175
150
125
100
75
50
25
0

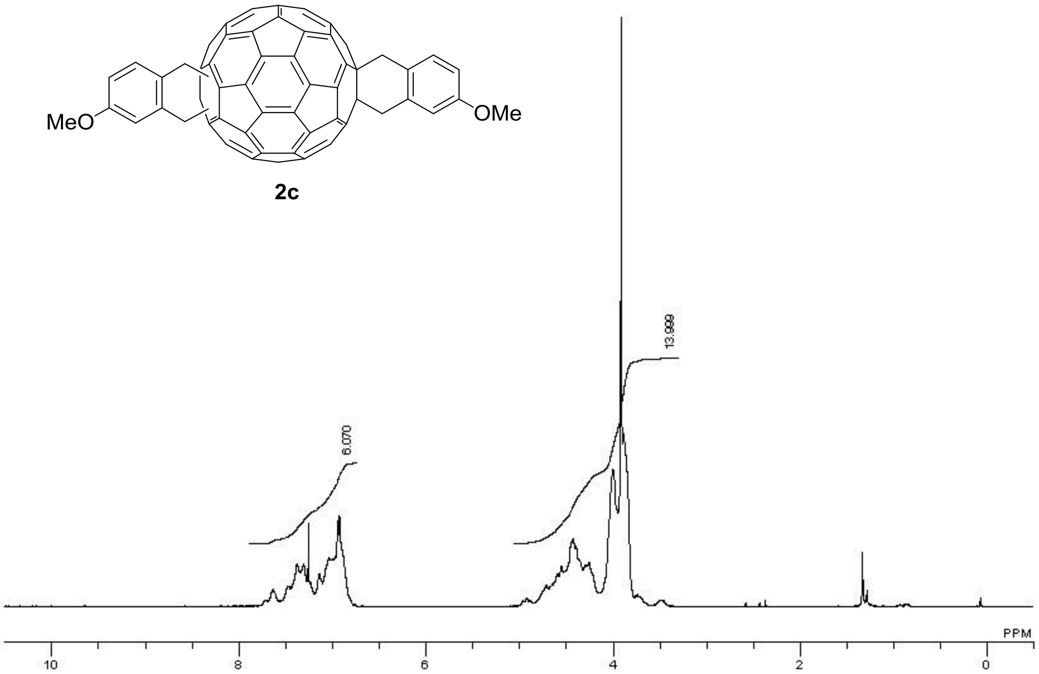
MeO
OMe
2c
13.999
6.070
PPM
10
8
6
4
2
0

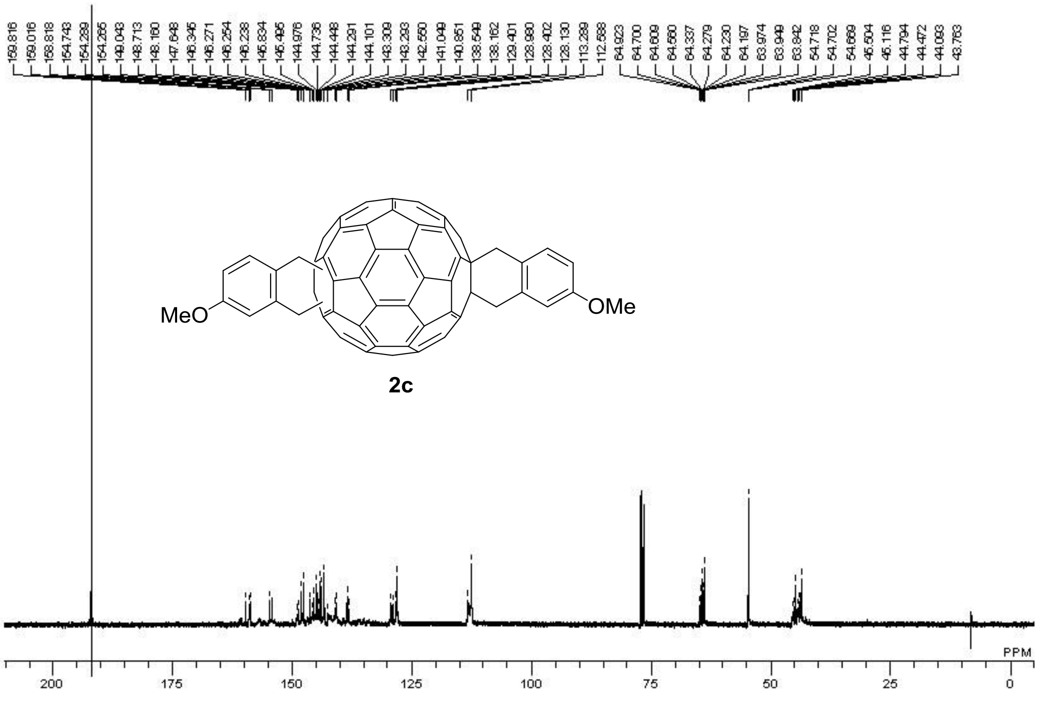
159.816
159.016
158.818
154.743
154.289
154.265
149.043
148.713
148.160
147.648
146.345
146.271
146.254
146.238
145.834
145.495
144.976
144.736
144.448
144.291
144.101
143.309
143.293
142.550
141.049
140.851
138.549
138.162
129.401
128.980
128.402
128.130
113.289
112.588
64.923
64.700
64.609
64.560
64.337
64.279
64.230
64.197
63.974
63.949
63.842
54.718
54.702
54.669
45.504
45.116
44.794
44.472
44.093
43.763
MeO
OMe
2c
PPM
200
175
150
125
100
75
50
25
0

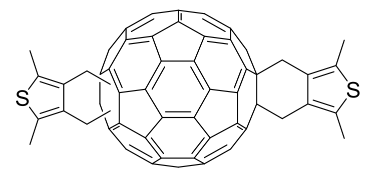

2d

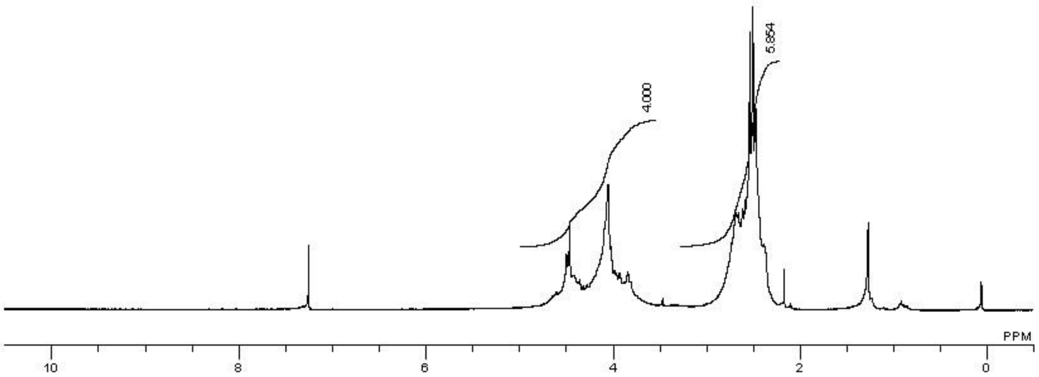
4.000
5.854
PPM
10
8
6
4
2
0

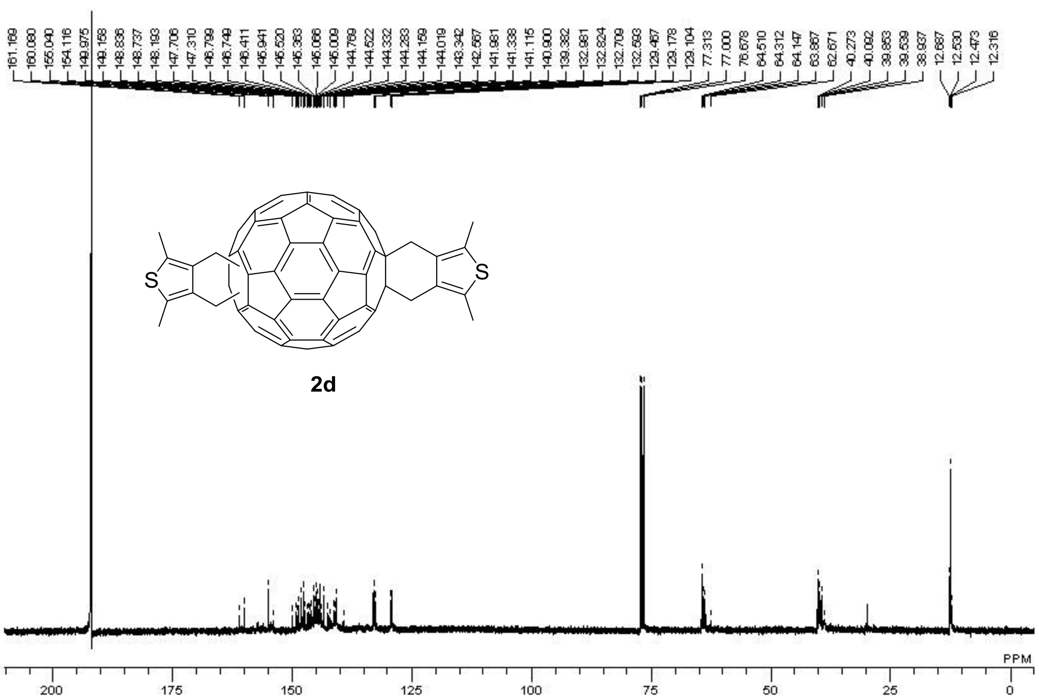
161.169 160.080 155.040 154.116 149.975 149.158 148.836 148.737 148.193 147.706 147.310 146.799 146.749 146.411 145.941 145.520 145.363 145.066 145.009 144.769 144.522 144.332 144.283 144.159 144.019 143.342 142.567 141.981 141.338 141.115 140.900 139.382 132.981 132.824 132.709 132.593 129.467 129.178 129.104 77.313 77.000 76.678 64.510 64.312 64.147 63.867 62.671 40.273 40.092 39.853 39.539 38.937 12.687 12.530 12.473 12.316
2d
PPM
200
175
150
125
100
75
50
25
0

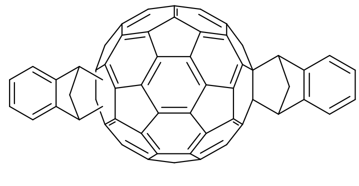

2e

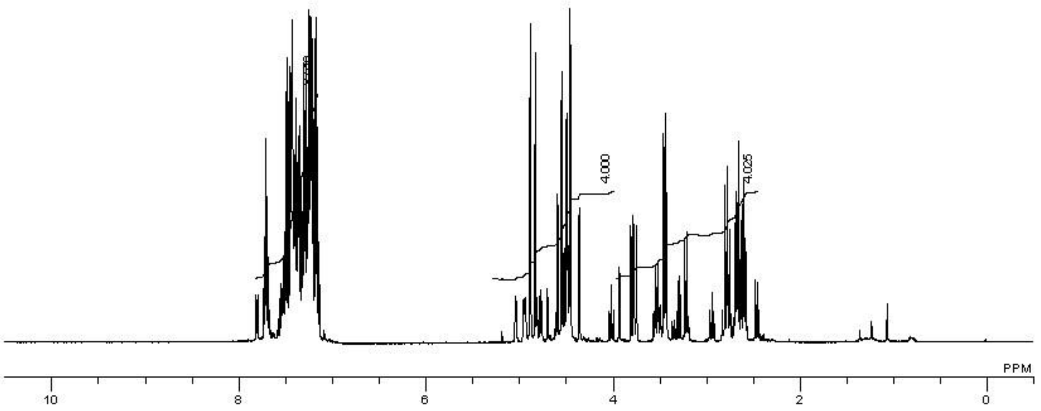
4.000
4.025
PPM
10
8
6
4
2
0

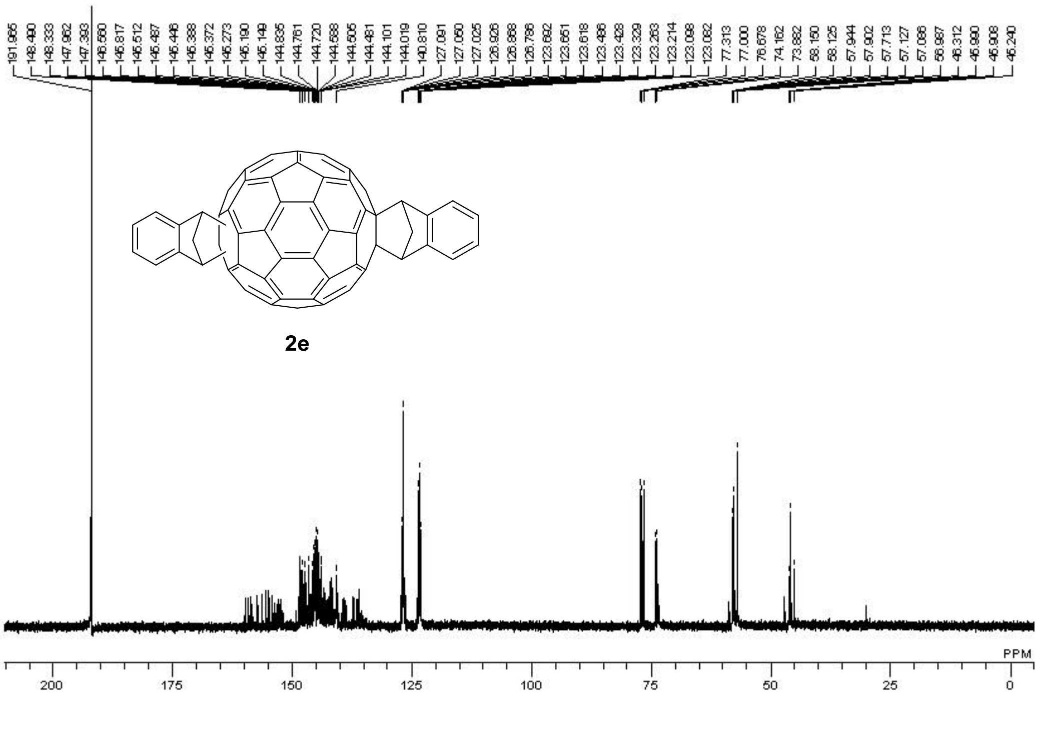
191.965
148.490
148.333
147.962
147.393
146.560
145.817
145.512
145.487
145.446
145.388
145.372
145.273
145.190
145.149
144.835
144.761
144.720
144.588
144.505
144.481
144.101
144.019
140.810
127.091
127.050
127.025
126.926
126.868
126.786
123.692
123.651
123.618
123.486
123.428
123.329
123.263
123.214
123.098
123.082
77.313
77.000
76.678
74.162
73.882
58.150
58.125
57.944
57.902
57.713
57.127
57.086
56.987
46.312
45.990
45.908
45.240
2e
PPM
200
175
150
125
100
75
50
25
0

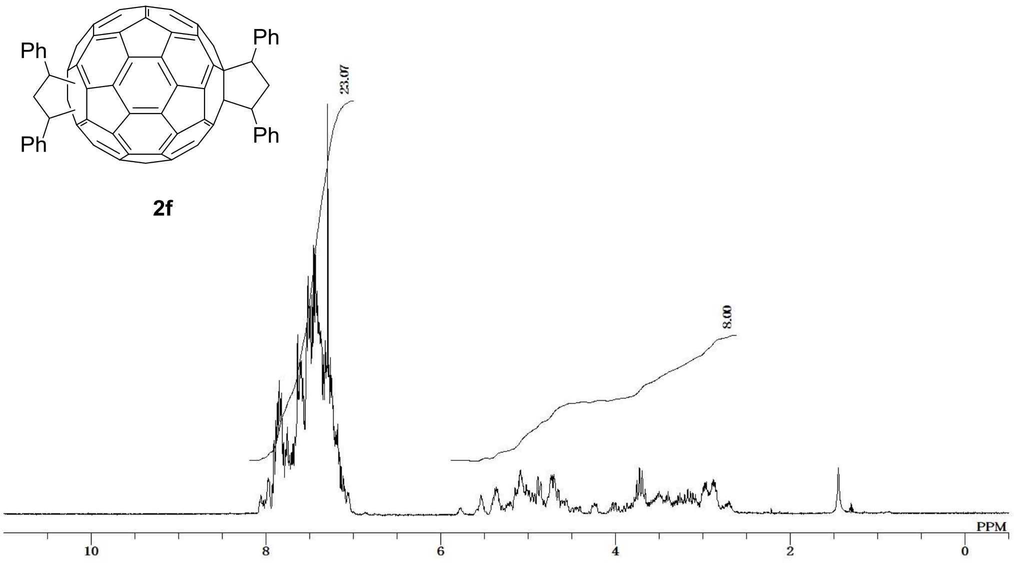
Ph
Ph
Ph
Ph
2f
23.07
8.00
PPM
10
8
6
4
2
0

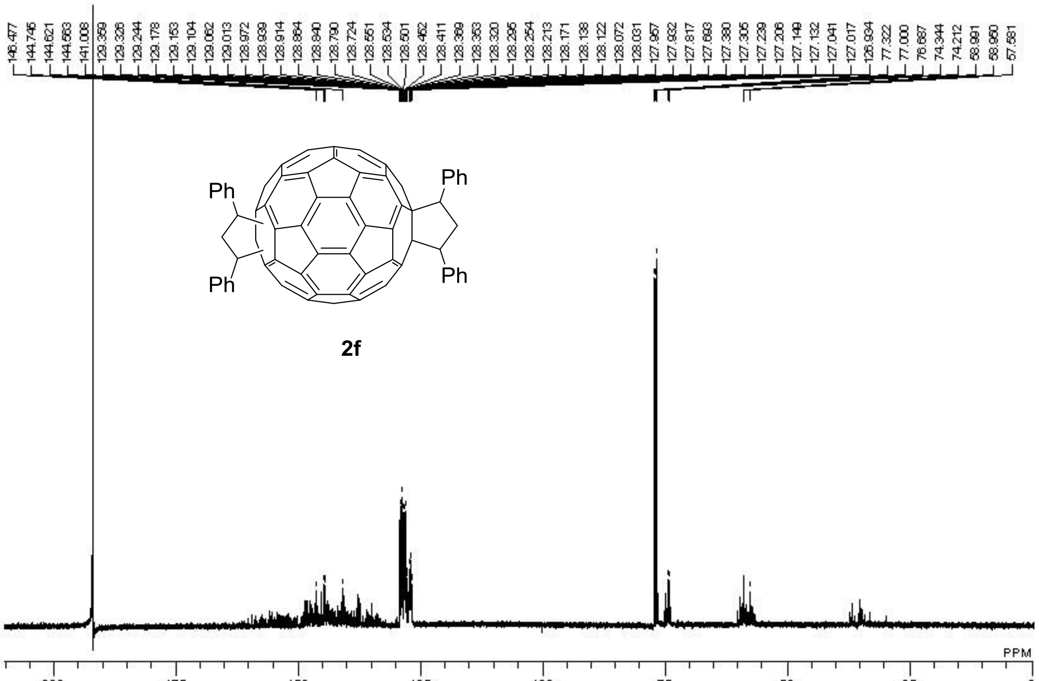
146.477
144.745
144.621
144.563
141.008
129.369
129.326
129.244
129.178
129.153
129.104
129.062
129.013
128.972
128.939
128.914
128.864
128.840
128.790
128.724
128.551
128.534
128.501
128.462
128.411
128.369
128.363
128.320
128.295
128.254
128.213
128.171
128.138
128.122
128.072
128.031
127.957
127.932
127.817
127.693
127.380
127.305
127.239
127.206
127.149
127.132
127.041
127.017
126.934
77.322
77.000
76.687
74.344
74.212
58.991
58.950
57.581
Ph
Ph
Ph
Ph
2f
PPM
200
175
150
125
100
75
50
25
0

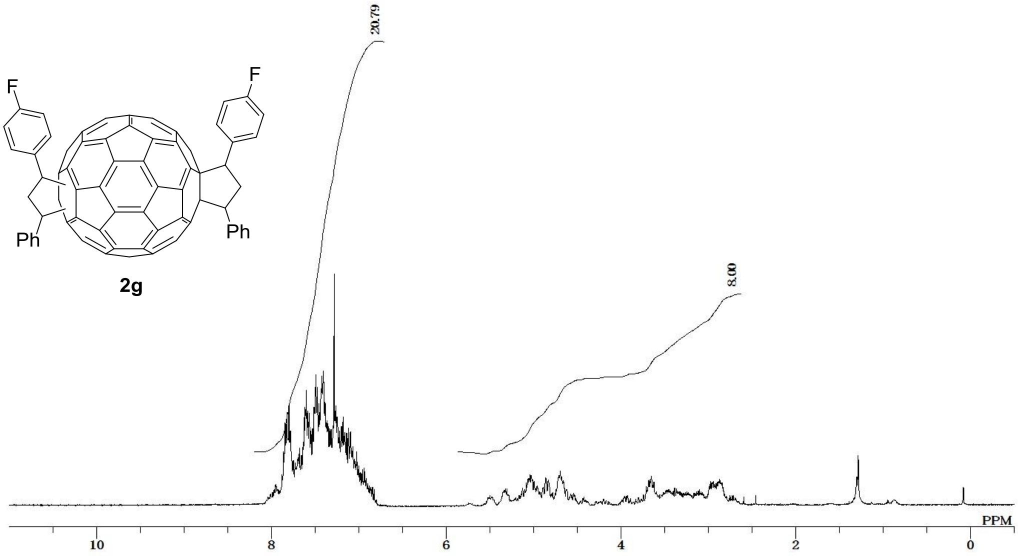
F
F
Ph
Ph
2g
20.79
8.00
PPM
10
8
6
4
2
0

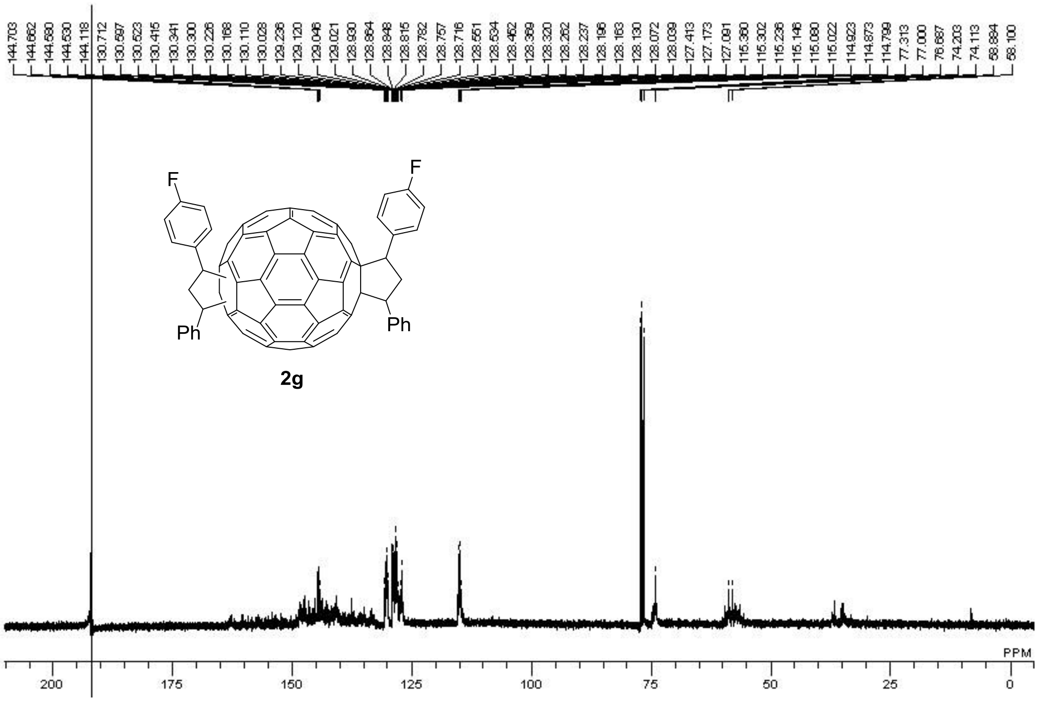
144.703
144.662
144.580
144.530
144.118
130.712
130.597
130.523
130.415
130.341
130.300
130.226
130.168
130.110
130.028
129.236
129.120
129.046
129.021
128.930
128.864
128.848
128.815
128.782
128.757
128.716
128.551
128.534
128.462
128.369
128.320
128.262
128.237
128.196
128.163
128.130
128.072
128.039
127.413
127.173
127.091
115.360
115.302
115.236
115.146
115.080
115.022
114.923
114.873
114.799
77.313
77.000
76.687
74.203
74.113
58.884
58.100
F
F
Ph
Ph
2g
PPM
200
175
150
125
100
75
50
25
0

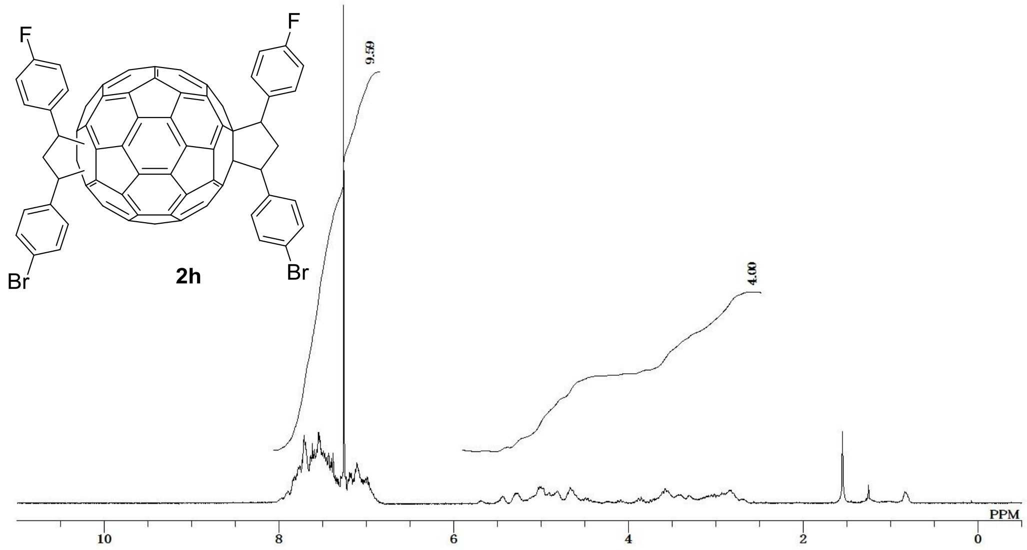
F
F
Br
2h
Br
9.59
4.00
PPM
10
8
6
4
2
0

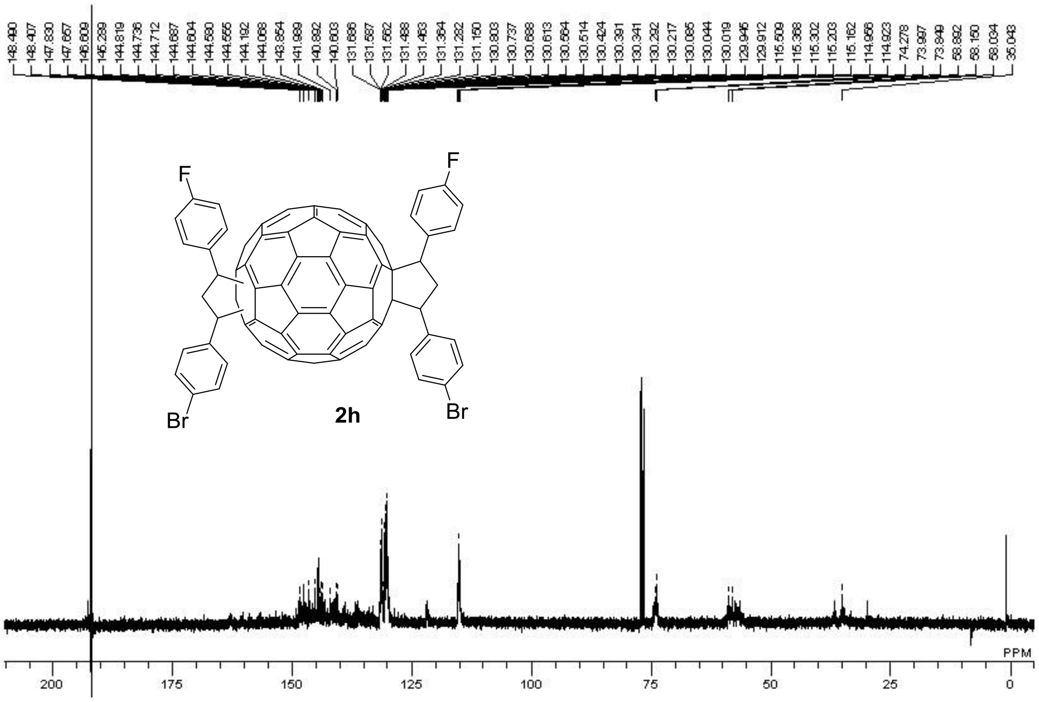
148.490
148.407
147.830
147.657
146.609
145.289
144.819
144.736
144.712
144.687
144.604
144.580
144.555
144.192
144.068
143.854
141.989
140.892
140.603
131.686
131.587
131.562
131.488
131.463
131.364
131.282
131.150
130.803
130.737
130.688
130.613
130.564
130.514
130.424
130.391
130.341
130.292
130.217
130.085
130.044
130.019
129.945
129.912
115.509
115.368
115.302
115.203
115.162
114.956
114.923
74.278
73.997
73.849
58.892
58.150
58.034
35.043
F
F
Br
2h
Br
PPM
200
175
150
125
100
75
50
25
0

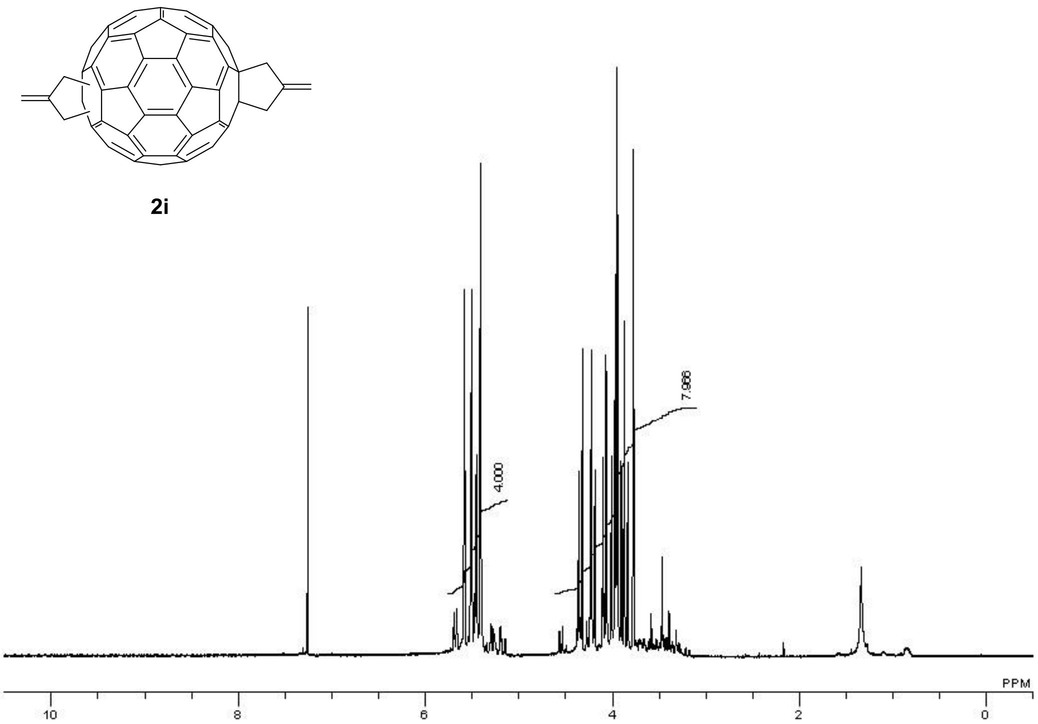
2i
4.000
7.966
PPM
10
8
6
4
2
0

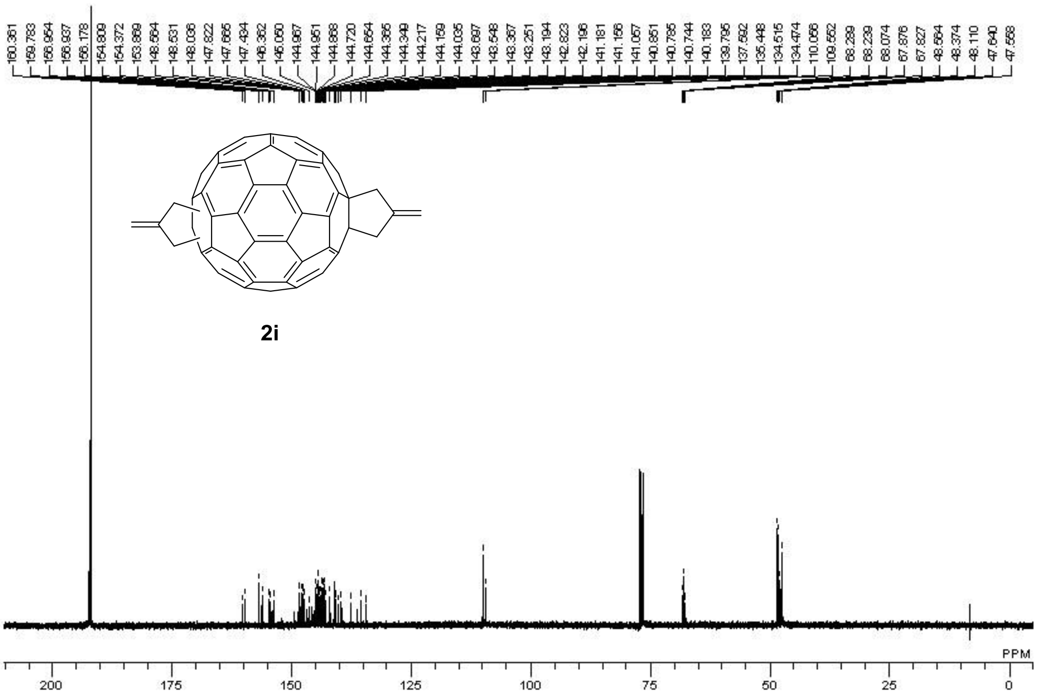
160.361
159.783
156.954
156.937
156.178
154.809
154.372
153.869
148.564
148.531
148.036
147.822
147.665
147.434
146.362
145.050
144.967
144.951
144.868
144.720
144.654
144.365
144.349
144.217
144.159
144.035
143.697
143.548
143.367
143.251
143.194
142.823
142.196
141.181
141.156
141.057
140.851
140.785
140.744
140.183
139.795
137.592
135.448
134.515
134.474
110.056
109.552
68.289
68.239
68.074
67.876
67.827
48.564
48.374
48.110
47.640
47.568
2i
PPM
200
175
150
125
100
75
50
25
0

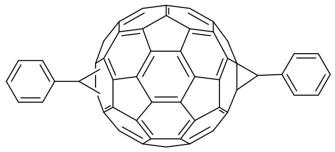

2j

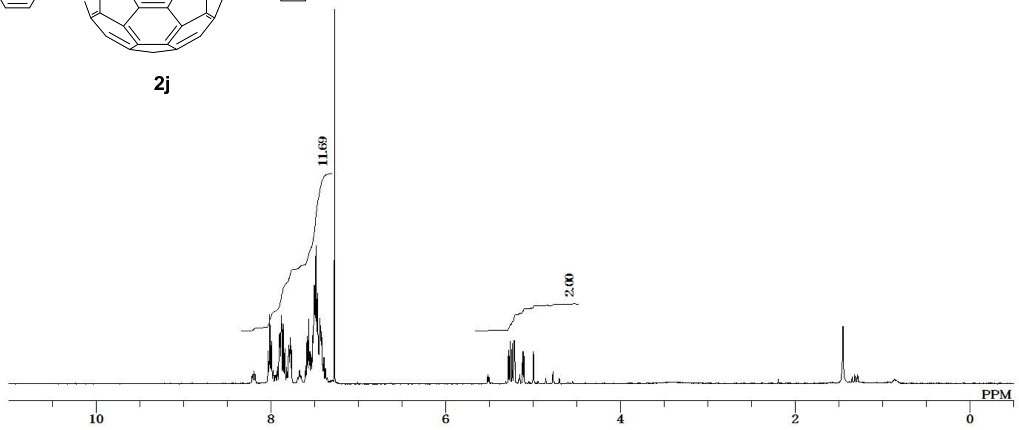
11.69
2.00
PPM
10
8
6
4
2
0

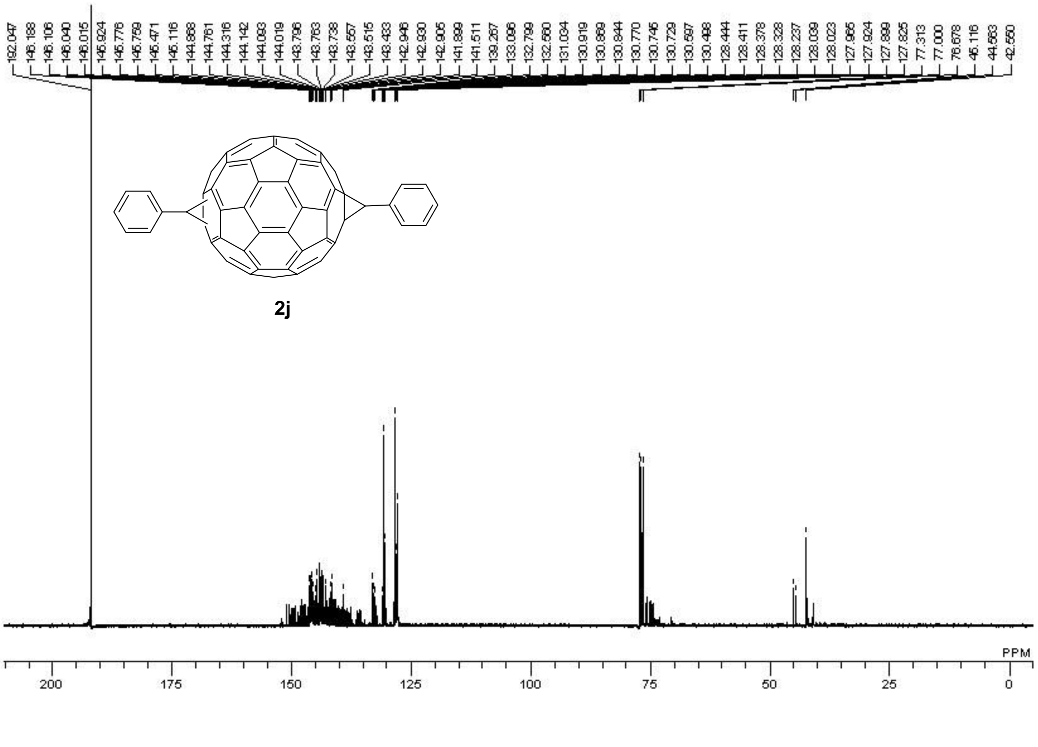
192.047
146.188
146.106
146.040
146.015
145.924
145.776
145.759
145.471
145.116
144.868
144.761
144.316
144.142
144.093
144.019
143.796
143.763
143.738
143.557
143.515
143.433
142.946
142.930
142.905
141.899
141.511
139.267
133.096
132.799
132.560
131.034
130.919
130.869
130.844
130.770
130.746
130.729
130.597
130.498
128.444
128.411
128.378
128.328
128.237
128.039
128.023
127.965
127.924
127.899
127.825
77.313
77.000
76.678
45.116
44.563
42.550
PPM
200
175
150
125
100
75
50
25
0

2j

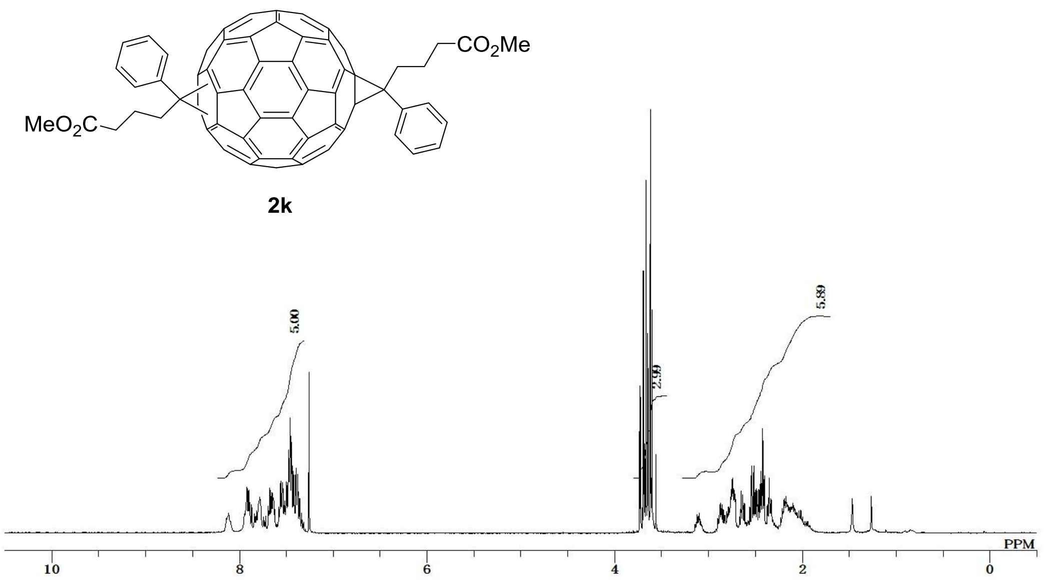

MeO2C
CO2Me
2k
5.00
2.99
5.89
PPM
10
8
6
4
2
0

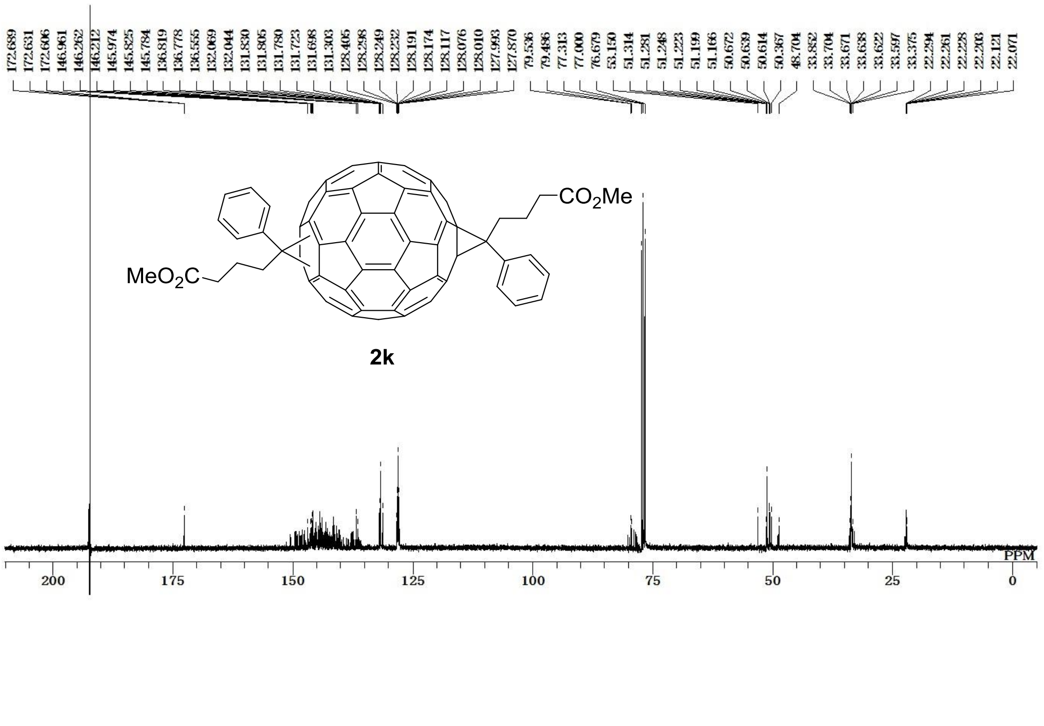

172.689 172.631 172.606 146.961 146.262 146.212 145.974 145.825 145.784 136.819 136.778 136.555 132.069 132.044 131.830 131.805 131.780 131.723 131.698 131.303 128.405 128.298 128.249 128.232 128.191 128.174 128.117 128.076 128.010 127.993 127.870 79.536 79.486 77.313 77.000 76.679 53.150 51.314 51.281 51.248 51.223 51.199 51.166 50.672 50.639 50.614 50.367 48.704 33.852 33.704 33.671 33.638 33.622 33.597 33.375 22.294 22.261 22.228 22.203 22.121 22.071
MeO2C
CO2Me
2k
PPM
200
175
150
125
100
75
50
25
0

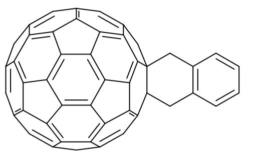

3a

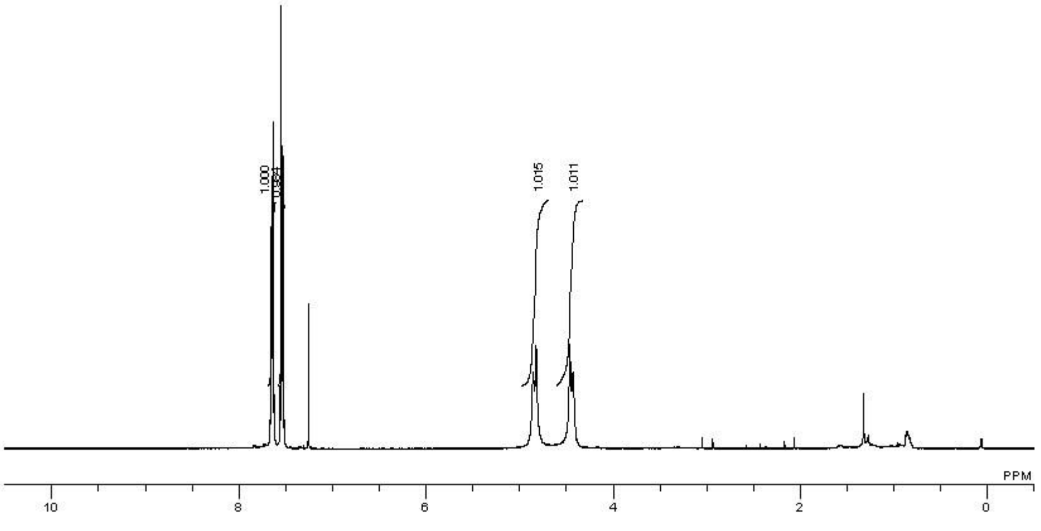

1.000
0.981
1.015
1.011
PPM
10
8
6
4
2
0

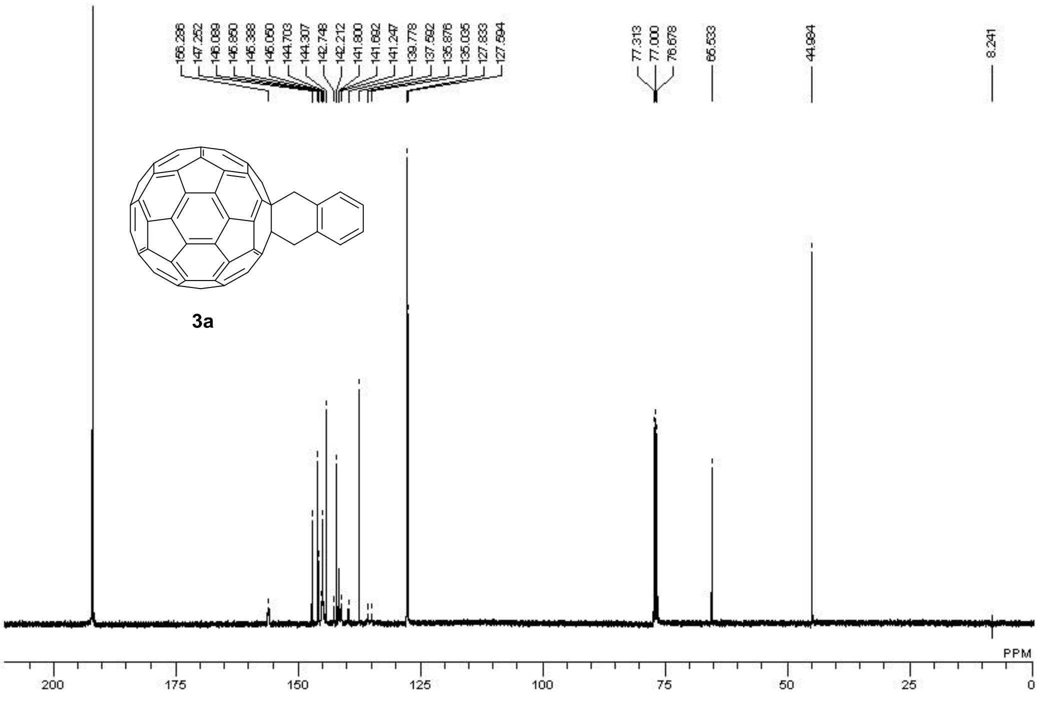

156.286
147.252
146.089
145.850
145.388
145.050
144.703
144.307
142.748
142.212
141.800
141.662
141.247
139.778
137.592
135.876
135.035
127.833
127.594
77.313
77.000
76.678
65.533
44.984
8.241
3a
PPM
200
175
150
125
100
75
50
25
0

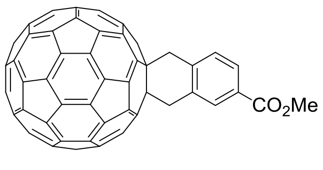

3b

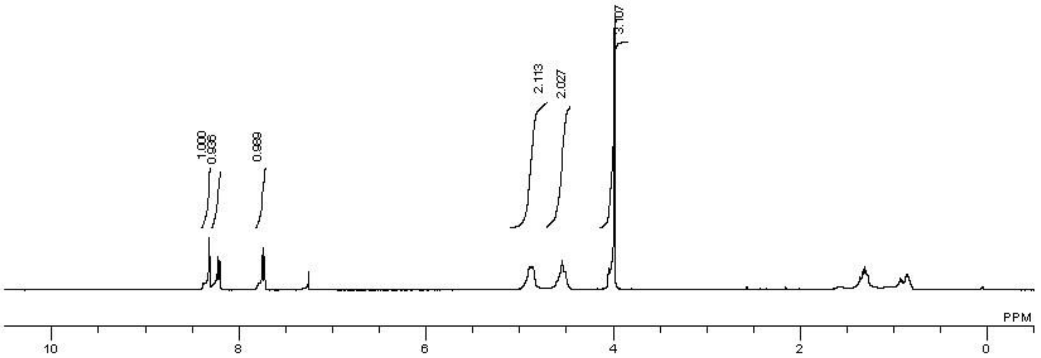

1.000
0.936
0.989
2.113
2.027
3.107
PPM
10
8
6
4
2
0

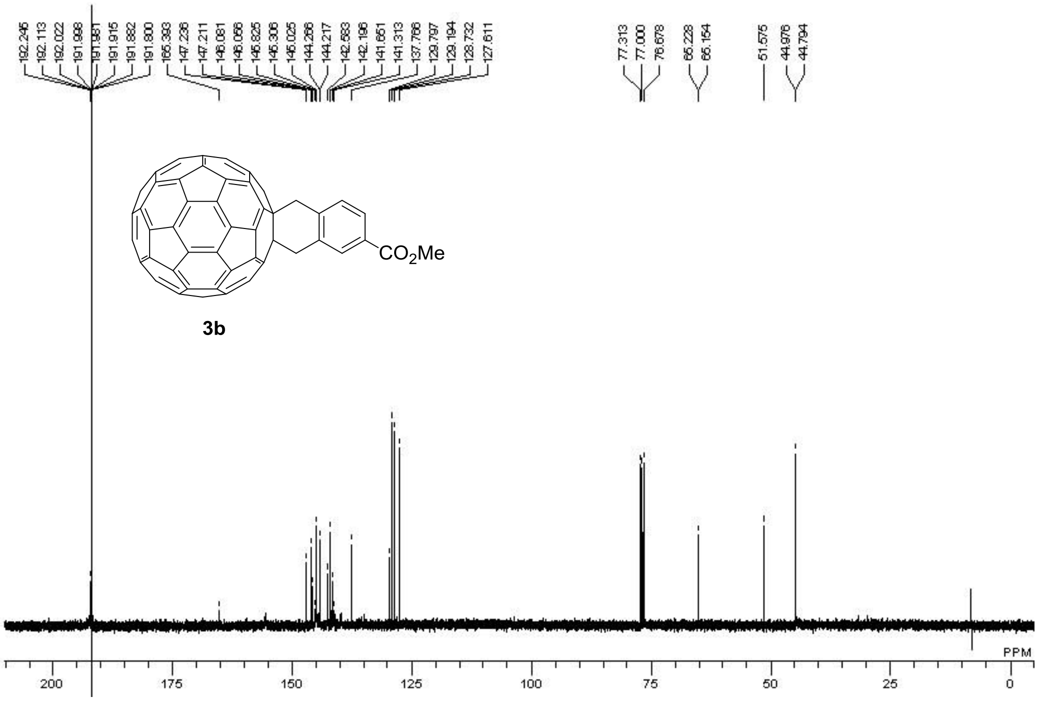

192.245
192.113
192.022
191.998
191.981
191.915
191.882
191.800
165.393
147.236
147.211
146.081
146.056
145.825
145.306
145.025
144.266
144.217
142.583
142.196
141.651
141.313
137.766
129.797
129.194
128.732
127.611
77.313
77.000
76.678
65.228
65.154
51.575
44.976
44.794
CO_2Me
3b
PPM
200
175
150
125
100
75
50
25
0

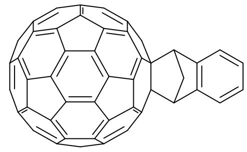

3c

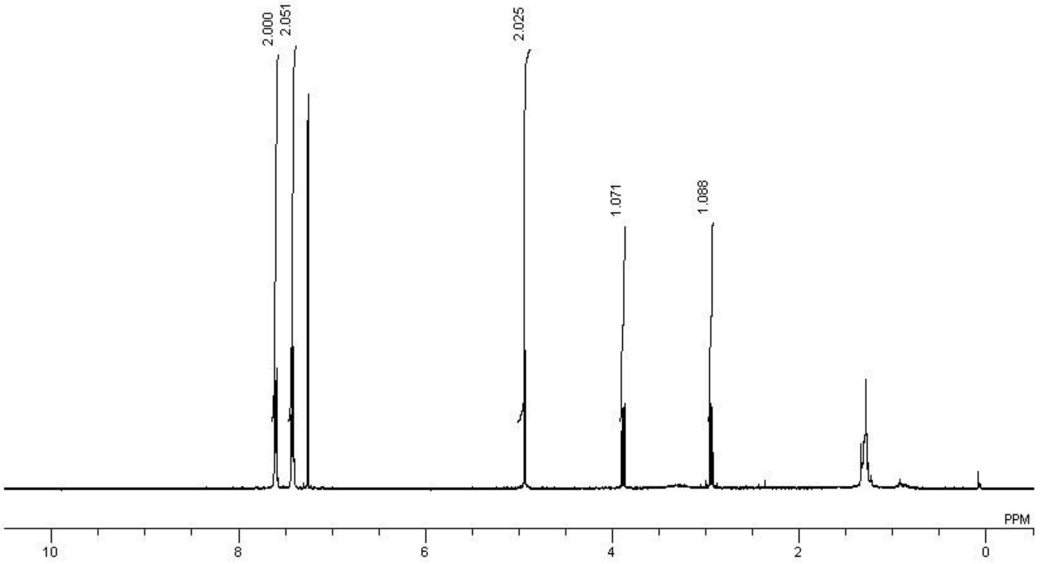
2.000
2.051
2.025
1.071
1.088
PPM
10
8
6
4
2
0

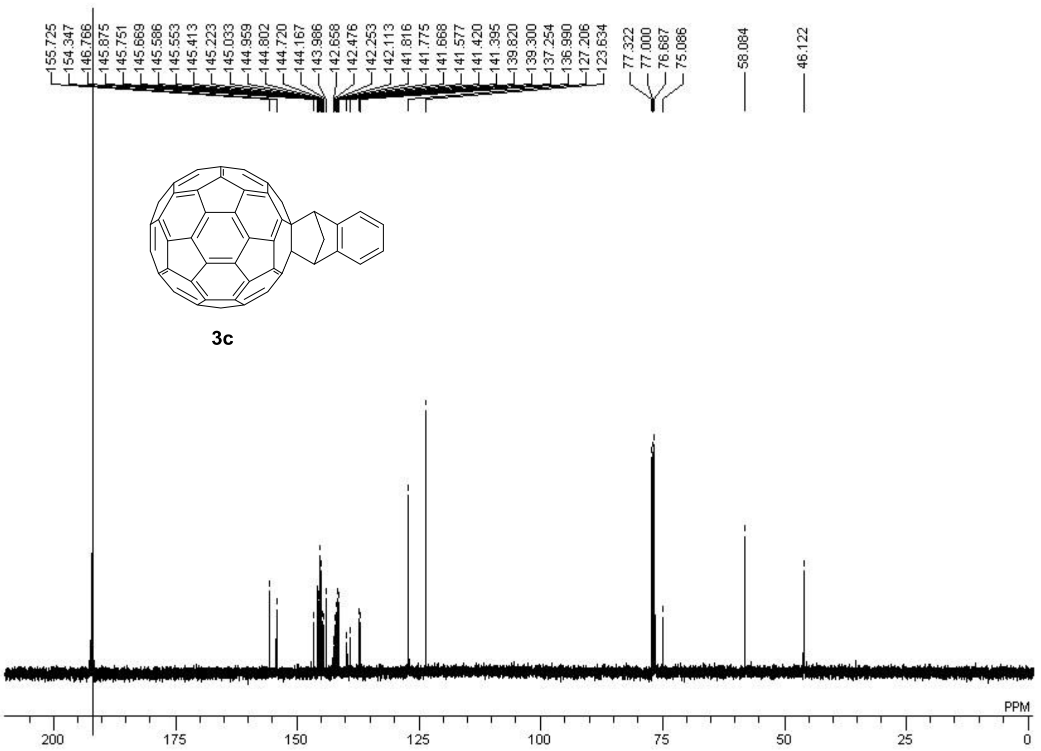
155.725
154.347
146.766
145.875
145.751
145.669
145.586
145.553
145.413
145.223
145.033
144.959
144.802
144.720
144.167
143.986
142.658
142.476
142.253
142.113
141.816
141.775
141.668
141.577
141.420
141.395
139.820
139.300
137.254
136.990
127.206
123.634
77.322
77.000
76.687
75.086
58.084
46.122
3c
PPM
200
175
150
125
100
75
50
25
0

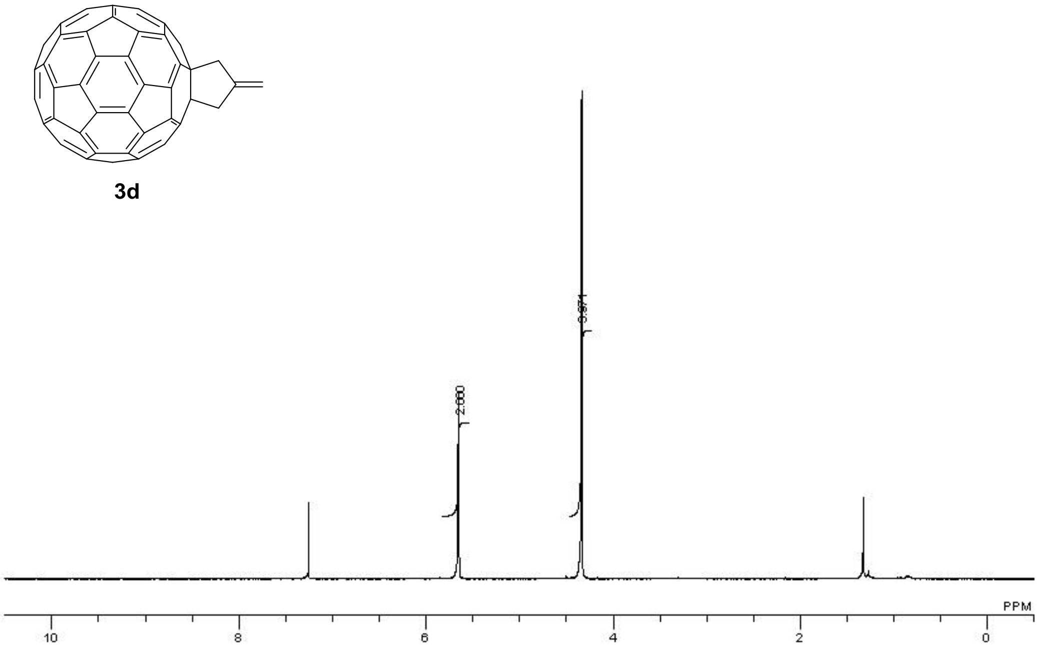
3d
2.000
3.971
PPM
10
8
6
4
2
0

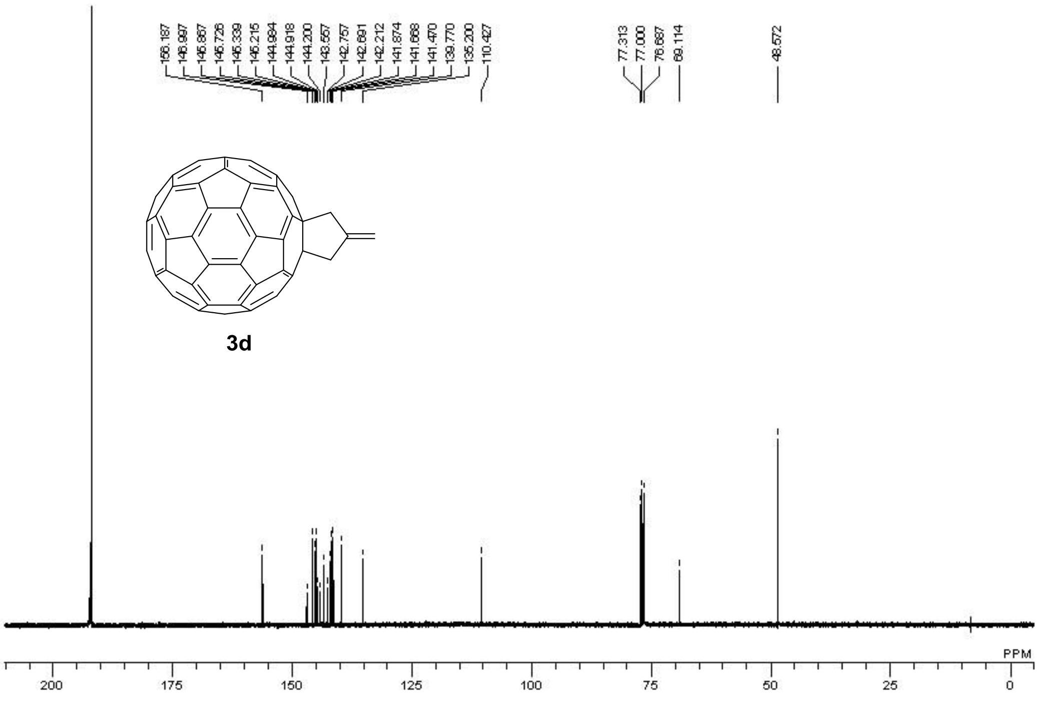
156.187
146.997
145.867
145.726
145.339
145.215
144.984
144.918
144.200
143.557
142.757
142.691
142.212
141.874
141.668
141.470
139.770
135.200
110.427
77.313
77.000
76.687
69.114
48.572
PPM
200
175
150
125
100
75
50
25
0
3d

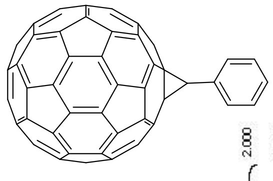

3e

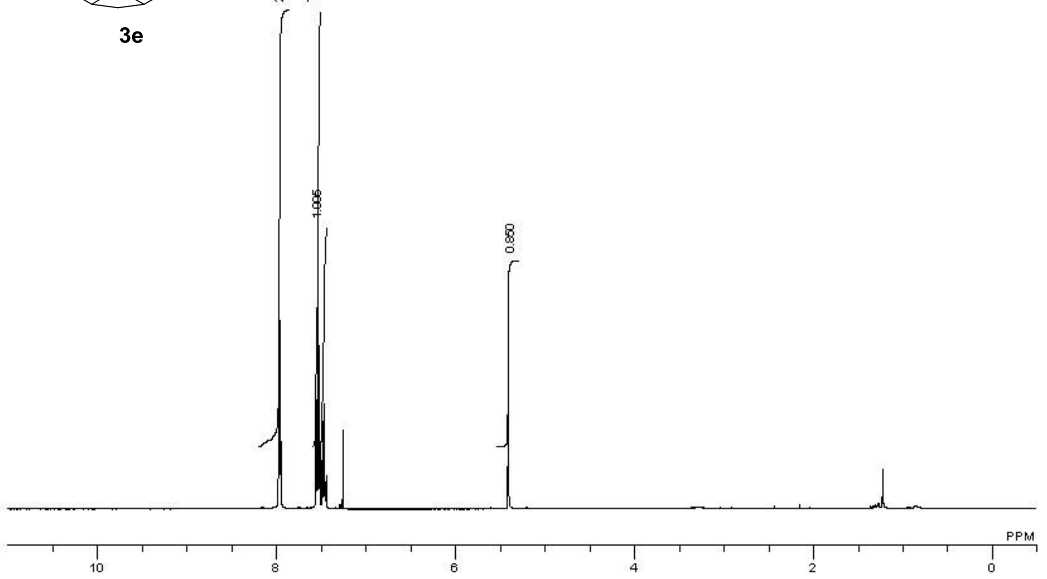

2.000
1.994
1.006
0.850
PPM
10
8
6
4
2
0

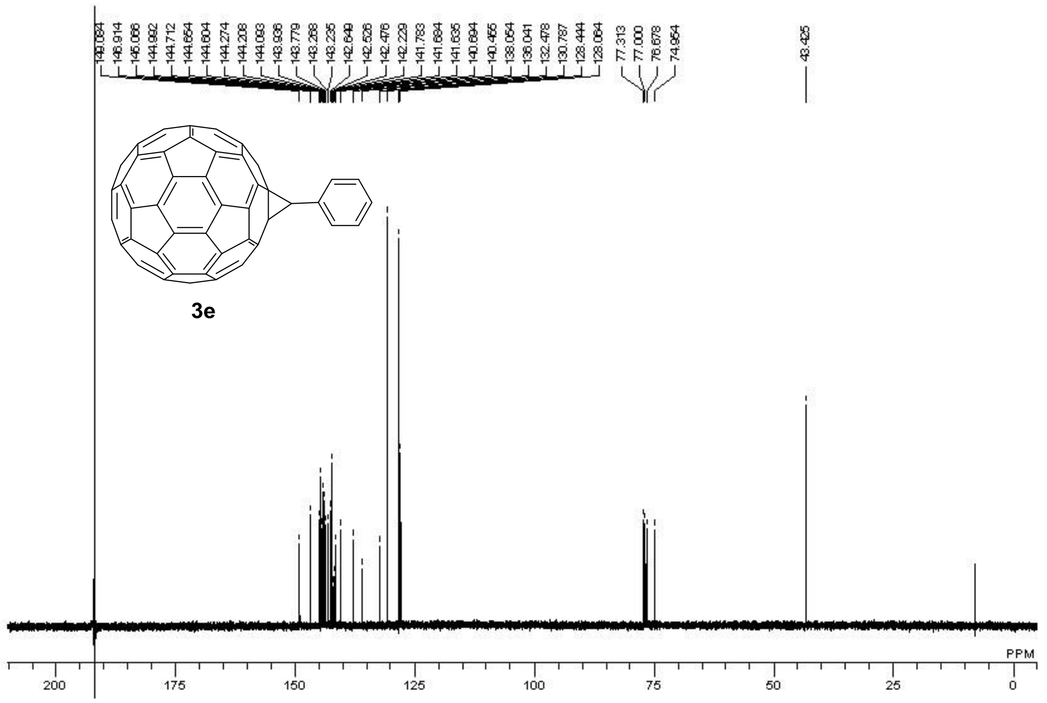

146.914
145.066
144.992
144.712
144.654
144.604
144.274
144.208
144.093
143.936
143.779
143.268
143.235
142.649
142.526
142.476
142.229
141.783
141.684
141.635
140.694
140.455
138.054
136.041
132.478
130.787
128.444
128.064
77.313
77.000
76.678
74.954
43.425
3e
PPM
200
175
150
125
100
75
50
25
0

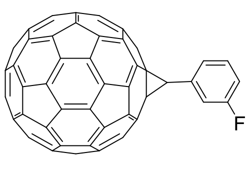

3f

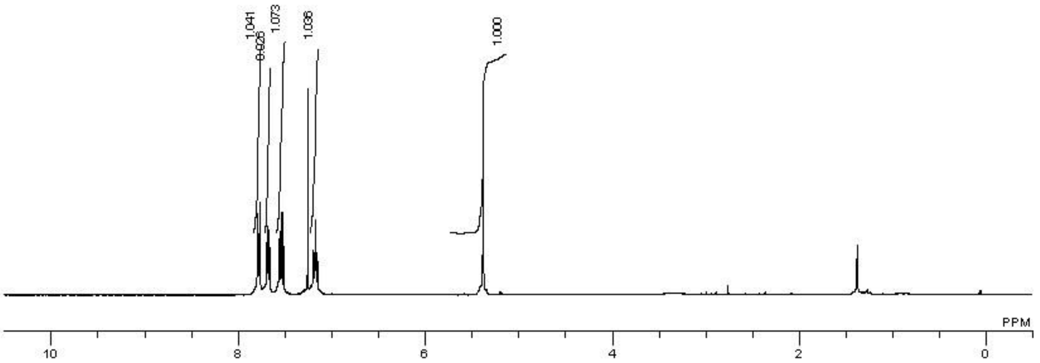
1.041
1.073
1.036
1.000
PPM
10
8
6
4
2
0

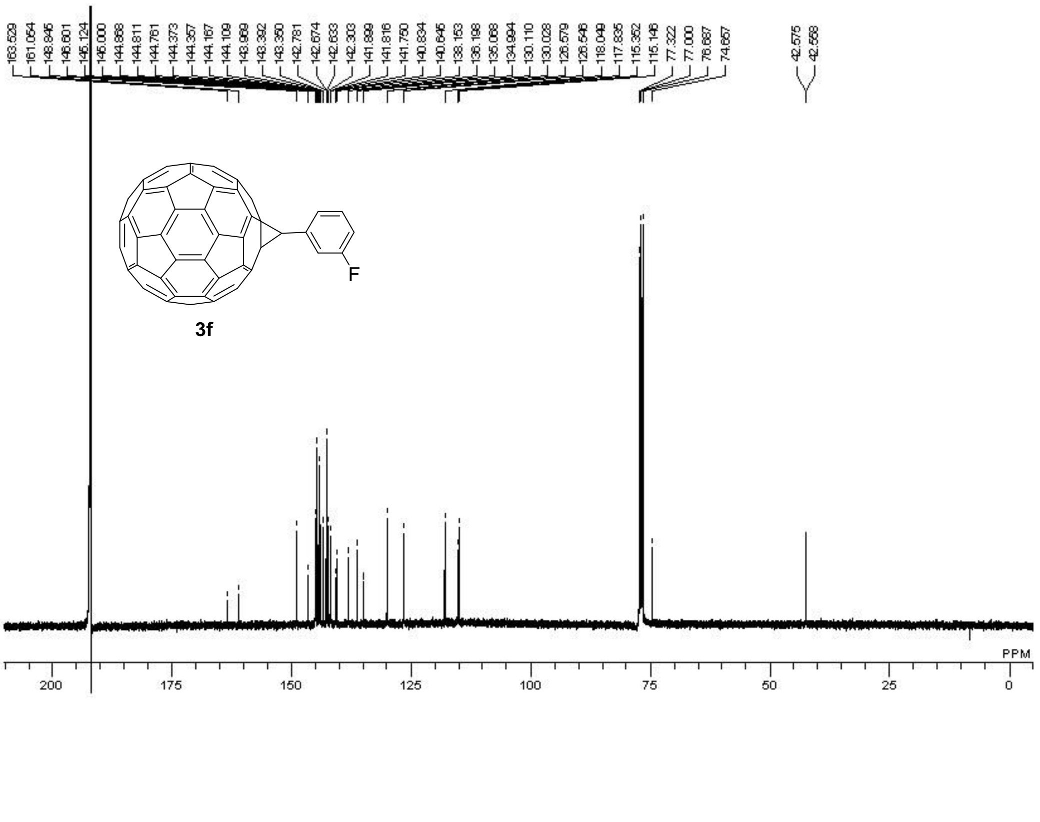
163.529
161.054
148.845
146.601
145.124
145.000
144.868
144.811
144.761
144.373
144.357
144.167
144.109
143.969
143.392
143.350
142.781
142.674
142.633
142.303
141.899
141.816
141.750
140.834
140.645
138.153
136.198
135.068
134.994
130.110
130.028
126.579
126.546
118.049
117.835
115.352
115.146
77.322
77.000
76.687
74.657
42.575
42.558
F
3f
PPM
200
175
150
125
100
75
50
25
0

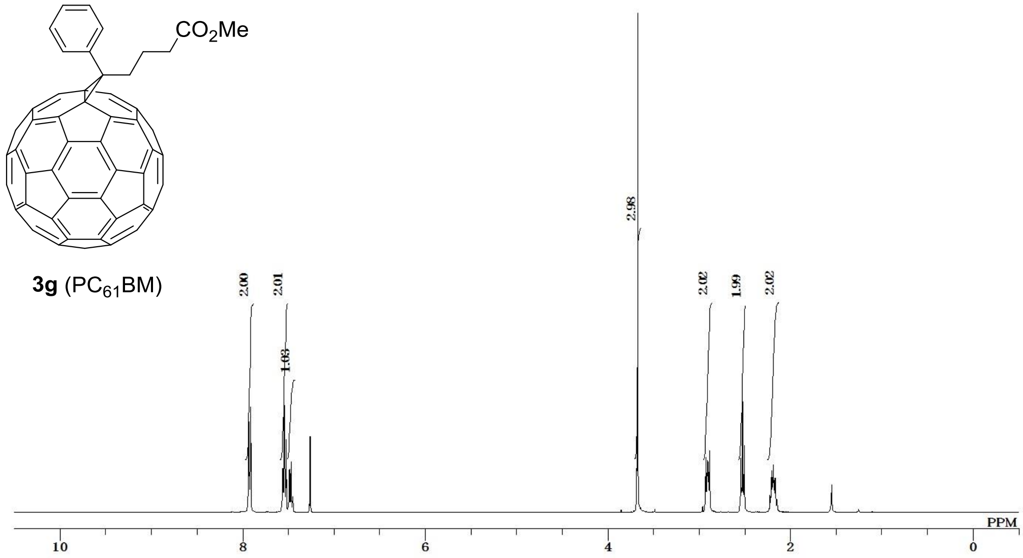
CO$_2$Me
3g (PC$_{61}$BM)
2.00
2.01
1.03
2.98
2.02
1.99
2.02
PPM
10
8
6
4
2
0

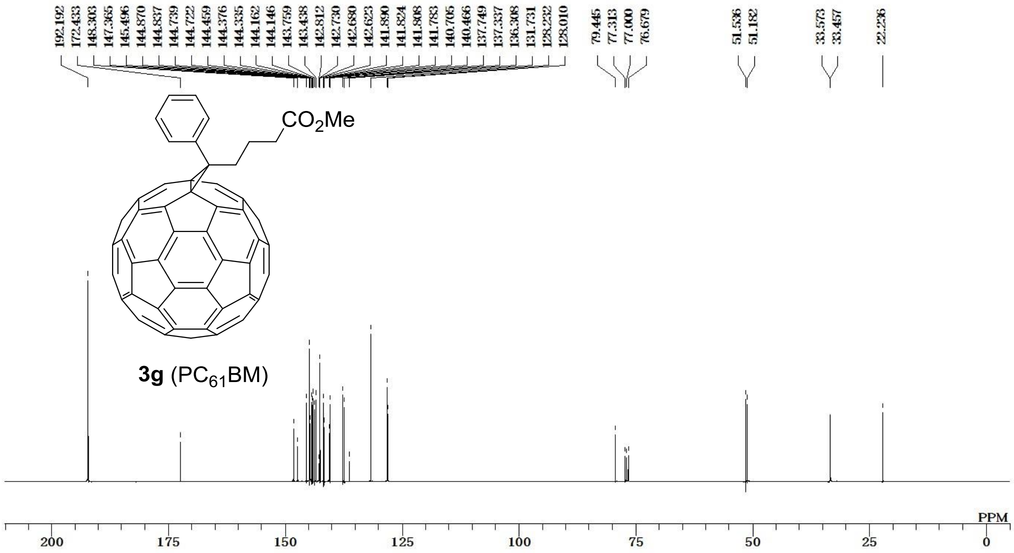
192.192
172.433
148.303
147.365
145.496
144.870
144.837
144.739
144.722
144.459
144.376
144.335
144.162
144.146
143.759
143.438
142.812
142.730
142.680
142.623
141.890
141.824
141.808
141.783
140.705
140.466
137.749
137.337
136.308
131.731
128.232
128.010
79.445
77.313
77.000
76.679
51.536
51.182
33.573
33.457
22.236
CO$_2$Me
3g (PC$_{61}$BM)
PPM
200
175
150
125
100
75
50
25
0

C:\NMR data\Yamamoto-Asao lab 2014 05 28\Si\Si-3\H-NMR\1583-PC71BM.als

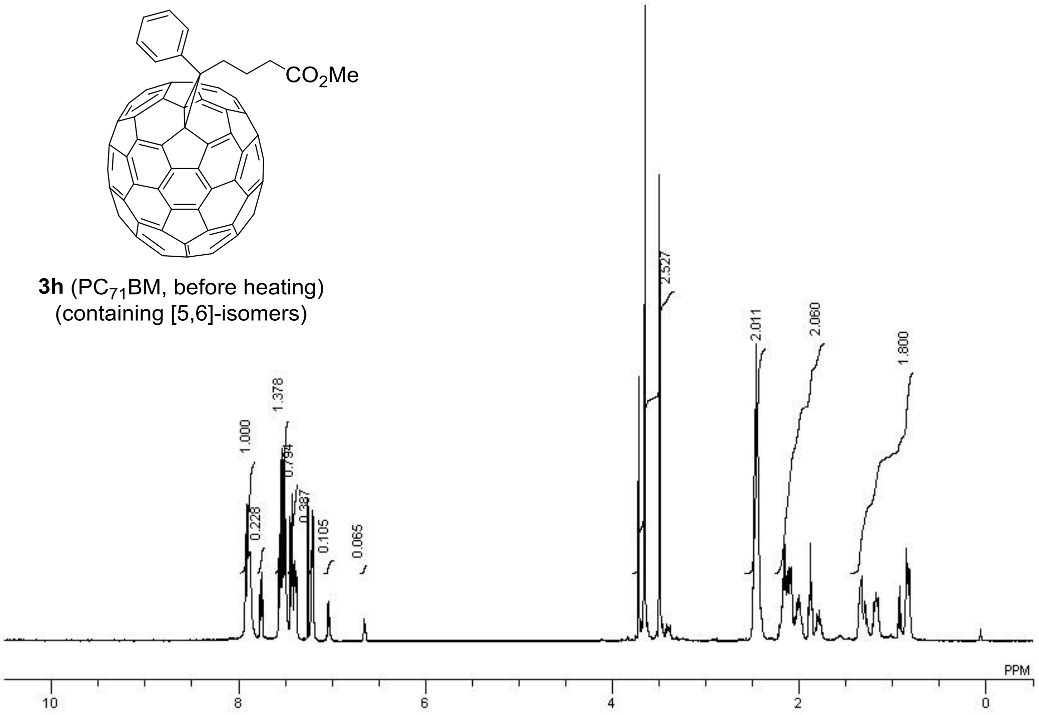

C:\NMR data\Yamamoto-Asao lab 2014 05 28\Si\Si-3\H-NMR\1583--13C-PC71BM.als

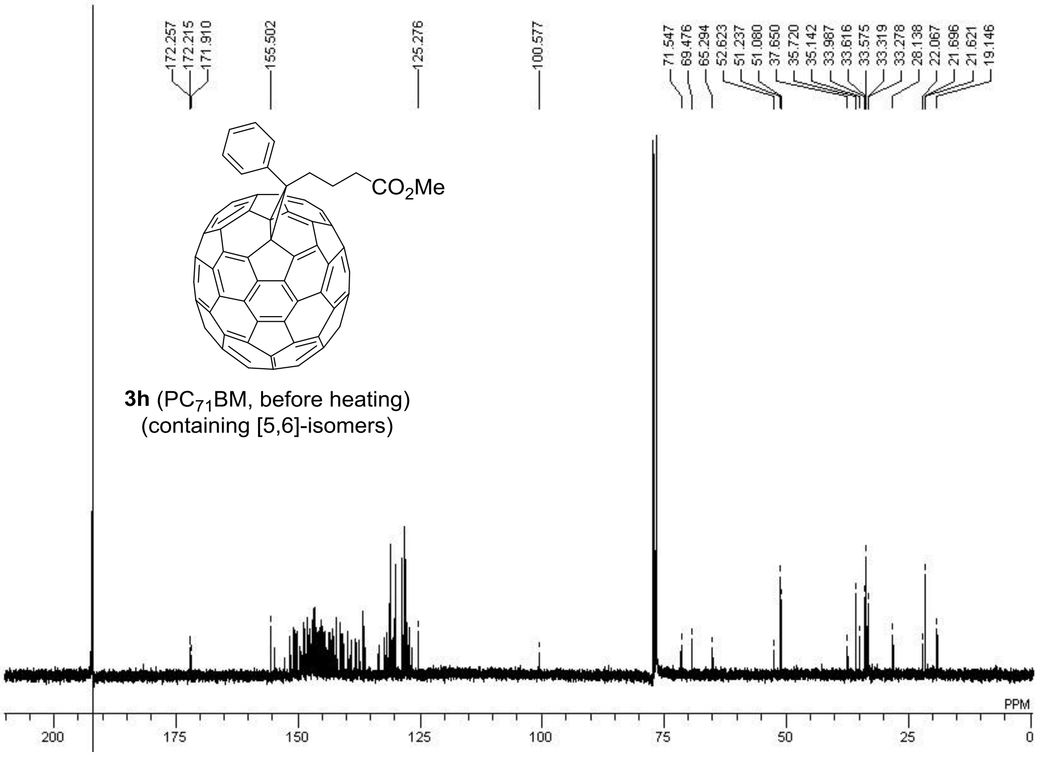

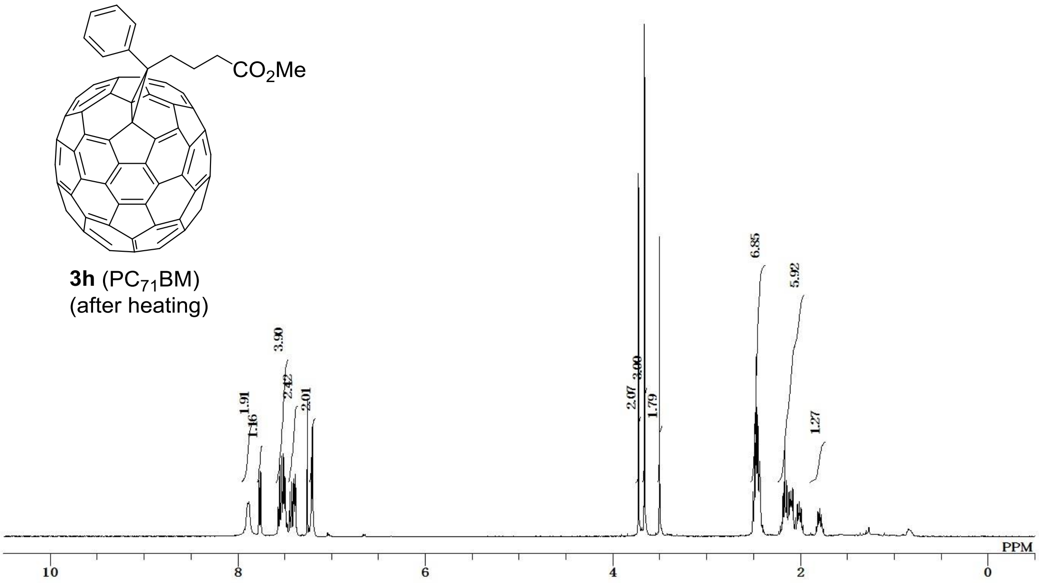
CO2Me
3h (PC71BM)
(after heating)
1.91
1.46
3.90
2.42
2.01
2.07
3.00
1.79
6.85
5.92
1.27
PPM
10
8
6
4
2
0

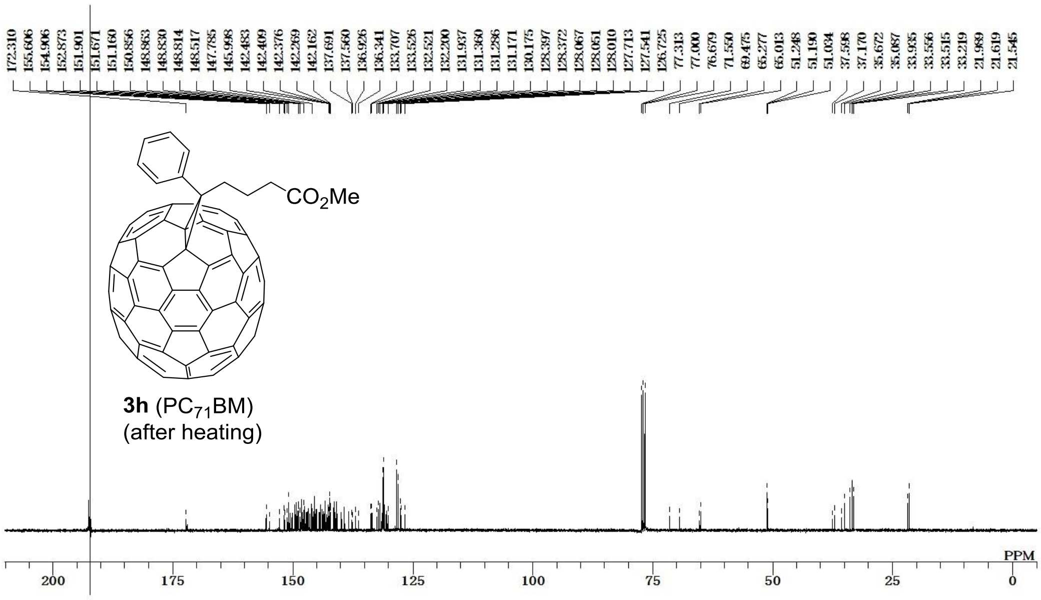
172.310
155.606
154.906
152.873
151.901
151.671
151.160
150.856
148.863
148.830
148.814
148.517
147.785
145.998
142.483
142.409
142.376
142.269
142.162
137.691
137.560
136.926
136.341
133.707
133.526
132.521
132.200
131.937
131.360
131.286
131.171
130.175
128.397
128.372
128.067
128.051
128.010
127.713
127.541
126.725
77.313
77.000
76.679
71.550
69.475
65.277
65.013
51.248
51.190
51.034
37.598
37.170
35.672
35.087
33.935
33.556
33.515
33.219
21.989
21.619
21.545
CO2Me
3h (PC71BM)
(after heating)
PPM
200
175
150
125
100
75
50
25
0
